# Analysis of Gene Expression in Experimental Pressure Ulcers in the Rat with Special Reference to Inflammatory Cytokines

**DOI:** 10.1371/journal.pone.0132622

**Published:** 2015-07-15

**Authors:** Tomoyuki Kurose, Masakazu Hashimoto, Junya Ozawa, Seiichi Kawamata

**Affiliations:** 1 Institute of Biomedical & Health Sciences, Hiroshima University, Hiroshima, Japan; 2 Faculty of Health Sciences, Hiroshima International University, Hiroshima, Japan; University of Leuven, Rega Institute, BELGIUM

## Abstract

Pressure ulcers have been investigated in a few animal models, but the molecular mechanisms of pressure ulcers are not well understood. We hypothesized that pressure results in up-regulation of inflammatory cytokines and those cytokines contribute to the formation of pressure ulcers. We measured genome-wide changes in transcript levels after compression, and focused especially on inflammatory cytokines. The abdominal wall of rats was compressed at 100 mmHg for 4 hours by two magnets. Specimens were obtained 12 hours, 1, or 3 days after compression, and analyzed by light microscopy, microarray, Real-Time PCR, and ELISA. The skin and subcutaneous tissue in the compressed area were markedly thickened. The microarray showed that numerous genes were up-regulated after the compression. Up-regulated genes were involved in apoptosis, inflammation, oxidative stress, proteolysis, hypoxia, and so on. Real-Time PCR showed the up-regulation of granulocyte-macrophage colony stimulating factor (GM-CSF), interferon γ (IFN-γ), interleukin 1β (IL-1β), interleukin 1 receptor antagonist gene (IL1Ra), interleukin 6 (IL-6), interleukin 10 (IL-10), matrix metalloproteinase 3 (MMP-3), tissue inhibitor of metalloproteinase 1 (TIMP-1), and tumor necrosis factor α (TNF-α) at 12 hours, IFN-γ, IL-6, IL-10, MMP-3, and TIMP-1 at 1 day, and IFN-γ, IL-6, and MMP-3 at 3 days. Some genes from subcutaneous tissue were up-regulated temporarily, and others were kept at high levels of expression. ELISA data showed that the concentrations of IL-1β and IL-6 proteins were most notably increased following compression. Prolonged up-regulation of IL-1β, and IL-6 might enhance local inflammation, and continuous local inflammation may contribute to the pressure ulcer formation. In addition, GM-CSF, IFN-γ, MMP-3, and TIMP-1 were not reported previously in the wound healing process, and those genes may have a role in development of the pressure ulcers. Expression data from Real-Time PCR were generally in good agreement with those of the microarray. Our microarray data were useful for identifying genes involved in pressure ulcer formation. However, the expression levels of the genes didn’t necessarily correspond with protein production. As such, the functions of these cytokines need to be further investigated.

## Introduction

Pressure ulcers, known as decubitus ulcers or decubitus sores, represent localized areas of tissue necrosis resulting from prolonged pressure. The ulcers are hard to heal, and treatment requires a lot of therapeutic agents. Patients pay the high cost of daily treatment until complete healing was achieved. Therefore, it may become a burden of medical expenses in various countries [1]. Factors causing pressure ulcers vary in patient’s situations, so identifying the main factor is so hard [2]. Because of this, an animal model is necessary in order to understand the mechanism. Previous animal studies reported that pressure-induced ischemia, friction, shear force, and reperfusion were important factors [3–9]. Recently, various disorders are widely studied by using molecular and biological techniques, but not so extensively concerning pressure ulcers. A microarray is a technique to provide comprehensive coverage of the transcribed genome, and is used to measure changes in expression levels simultaneously. To obtain genome-wide changes in pressure ulcer formation, we modified the magnetic compression model [9] and performed an Affymetrix DNA microarray to screen major genes that potentially play pivotal roles in the formation of pressure ulcers. Inflammatory cytokines were reported to contribute various tissue damages, so we hypothesized that inflammatory cytokines are up-regulated after compression of the skin.

## Materials and Methods

A total of 36 male Wistar rats (266.4 ± 20.8 g) aged 8 and 9 weeks were used. Animals were divided into a sham group and a compression group. The experimental procedures were approved by the Committee of Research Facilities for Laboratory Animal Science, Natural Science Center for Basic Research and Development, Hiroshima University.

### Compression group

The abdominal wall was compressed at 100 mmHg for four hours as previously described [9]. The rats were anesthetized and a neodymium magnet (25 × 20 × 2 mm, NeoMag, Ichikawa, Japan) was inserted into the peritoneal cavity. Then, another neodymium magnet (25 × 20 × 5 mm, NeoMag) was applied on the skin surface. Magnets were removed after compression. The rats (n = 7 in each group) were euthanized via overinhalation of diethyl ether for sampling at 12 hours, 1 day or 3 days after the start of compression. The skin and the subcutaneous tissue from 4 out of 7 rats in each group were removed from the abdominal wall, and prepared for light microscopy, mRNA analysis, and ELISA. One of the four samples in each group mentioned above was used for microarray analysis. Histologically, the subcutaneous tissue had severe damage, so we also analyzed only the subcutaneous tissue. Specimens containing only subcutaneous tissues were removed from the abdominal wall of 3 rats, and were analyzed by mRNA analysis.

### Sham group

A magnet was inserted into the peritoneal cavity, but the compression was not done. Rats were sacrificed at the same time points of the control group (n = 5 in each group). A comparable part of the abdominal wall as in the compression group was removed. In addition, one of the four samples in each group mentioned above was used for microarray analysis. Specimen containing only subcutaneous tissues were also removed from the abdominal wall of one rat.

### Light microscopy

The rostral half of all specimens were trimmed and coated with 6% tragacanth gum jelly [9], [10]. The specimens were frozen in isopentane cooled by liquid nitrogen. Transverse sections (10 μm in thickness) were cut, stained with hematoxylin and eosin, and observed with an Olympus B51 light microscope (Olympus, Tokyo, Japan).

### RNA Isolation

The skin and the subcutaneous tissue at the central part of the compressed tissue were dissected into small pieces, and then immediately immersed in RNA*later* RNA Stabilization Reagent (Qiagen, Venlo, The Netherlands). RNA was extracted by using RNeasy Fibrous Tissue Mini Kit (Qiagen). RNA was assessed with spectrophotometry (UVmini-1240, Shimadzu Corp, Kyoto, Japan) and agarose gel electrophoresis.

### Microarray Analysis

RNA was assessed with an Agilent 2100 Bioanalyzer (Agilent Technologies, CA, U.S.A). Double-stranded cDNA was synthesized from total RNA. An in vitro transcription was performed to produce biotin-labeled cRNA from cDNA. Finally, cRNA was hybridized to a GeneChip Rat Genome 230 2.0 Array (Affymetrix, CA, U.S.A) for 16 hours at 45°C. This microarray chip provides the entire transcribed rat genome on a single array, and analyzes more than 31,000 genes at one time. After hybridization, chips were washed and dried, and then scanned by the GeneChip Scanner 3000 (Affymetrix). To calculate the fold change, the expression value of each gene from the compression group was divided by the expression value from the sham group. The whole microarray working process was accomplished at the Analysis Center of Life Science, Hiroshima University. Biological information of each probe was obtained from NetAffx Analysis Center (http://www.affymetrix.com/analysis/index.affx).

### Real-Time PCR (Real-Time Polymerase Chain Reaction)

Severe inflammation is assumed to break local tissues [11–13]. Thus we investigated the genes associated with an inflammatory response referring to Feldmann and Saklatvala [14].

Inflammatory cytokines were analyzed by Real-Time PCR, because Real-Time PCR may be more reliable than microarray analysis in quantification. For the synthesis of cDNA, 1 μg of RNA from each sample was reverse-transcribed by using the High Capacity cDNA Reverse Transcription Kit with RNase Inhibitor (Applied Biosystems, Foster City, CA, U.S.A). This kit contains Random primer, and all RNA in each solution is reverse-transcribed. Reverse transcription was initiated at 25°C for 10 minutes followed by 37°C for 2 hours, and at 85°C for 5 seconds with a Applied Biosystems 2720 Thermal Cycler (Applied Biosystems). One μl aliquots of the synthesized cDNA were added to 19 μl PCR mixture containing TaqMan Gene Expression Master Mix (4369016, Applied Biosystems) and TaqMan Gene Expression Assays (4331182, Applied Biosystems). The TaqMan Gene Expression Master Mix is a mixed component to perform Real-Time PCR without primers, probes, sample template, and water. The TaqMan Gene Expression Assays use a mixture of primers and fluorochrome (FAM or VIC)-labeled probes. TaqMan Gene Expression Assays associated with an inflammatory response (Table 1) were purchased. The relative quantity of the specific RNA in each sample was normalized to the endogenous reference β-actin. The mixed volume was kept at 50°C for 2 minutes to activate of UDG, which causes the release of uracil and blocking of DNA replication. After then, samples were incubated at 95°C for 10 minutes to activate the polymerase. The amplification conditions were 40 cycles at 95°C for 15 seconds and 60°C for 30 seconds. Real-Time PCR was performed with a QPCR system (Mx3000P, Stratagene, CA, U.S.A). Triplicate PCR reactions were measured for each sample. Data were analyzed by optional software MxPro Ver.4.0. The threshold line for quantification was determined by the software program automatically. To calculate the fold change, the expression value of each gene from the compression group was divided by the average expression value from the sham group.

**Table 1:** A list of Taqman Assay kits used for Real-time PCR.

Gene Symbol	Gene Name	Assay ID
β-actin	beta-actin	Rn00667869_m1
GM-CSF	granulocyte-macrophage colony stimulating factor	Rn01456851_m1
INF-γ	interferon gamma	Rn00594078_m1
IL-1α	interleukin 1 alpha	Rn00566700_m1
IL-1β	interleukin 1 beta	Rn00580432_m1
IL-1Ra	interleukin 1 receptor antagonist gene	Rn00573488_m1
IL-2	interleukin 2	Rn00587673_m1
IL-6	interleukin 6	Rn00561420_m1
IL-10	interleukin 10	Rn00563409_m1
MMP-3	matrix metalloproteinase 3	Rn00591740_m1
TGF-β1	transforming growth factor, beta-1	Rn00572010_m1
TIMP-1	tissue inhibitor of metalloproteinase 1	Rn00587558_m1
TNF-α	tumor necrosis factor, alpha	Rn99999017_m1

### Enzyme-linked immunosorbent assay (ELISA)

The skin and the subcutaneous tissue at the caudal part of a compressed area were dissected into small pieces, and then immediately immersed in PBS with protease inhibitor cocktail (P8340, Sigma, U.S.A). The total protein content was determined using a Coomassie Bradford Protein Assay Kit (23200, Pierce Biotechnology Inc., IL, U.S.A) with bovine serum albumin supplied as standard. Inflammatory cytokines (IFN-γ: ER-IFNG, IL-1α: ER-IL1A, IL-1β: ER2-IL1B. IL-6: ER2-IL6, IL-10: ER-IL10, TNF-α: ER3-TNFA, Pierce Biotechnology Inc.) were measured by an ELISA kit according to manufacturer’s instructions. The absorbance was measured at 450 nm in an automatic microplate reader (MTP-32, Corona Electric Co. Ibaraki, Japan). Data were analyzed by the optional software MTP-SF5 Ver.1.5.13. To calculate the fold change, the density of each cytokine from the compression group was divided by the average density from the sham group.

### Statistical Analysis

Statistical analysis was performed using Excel 2010 (Microsoft, Seattle, WA) with the add-in software Statcel 3 [15]. The *F*-test was used to assess the data of the expression levels from each group, and the values were homoscedastic. Therefore, the values were compared between experimental and sham group by Student’s *t*-test. Values of p < 0.05 were considered statistically significant.

## Results

Macroscopic and light microscopic findings are generally in agreement with Hashimoto *et al*. [9]. Macroscopically, the compression area was depressed, when the magnet laid on the skin was detached after compression. At 12 hours, edema and redness were observed. Edema and redness were gradually reduced, and the compression site was indistinguishable from the surrounding area. In the sham group, no changes were observed during the examined period.

### Light microscopy

**Fig 1:**
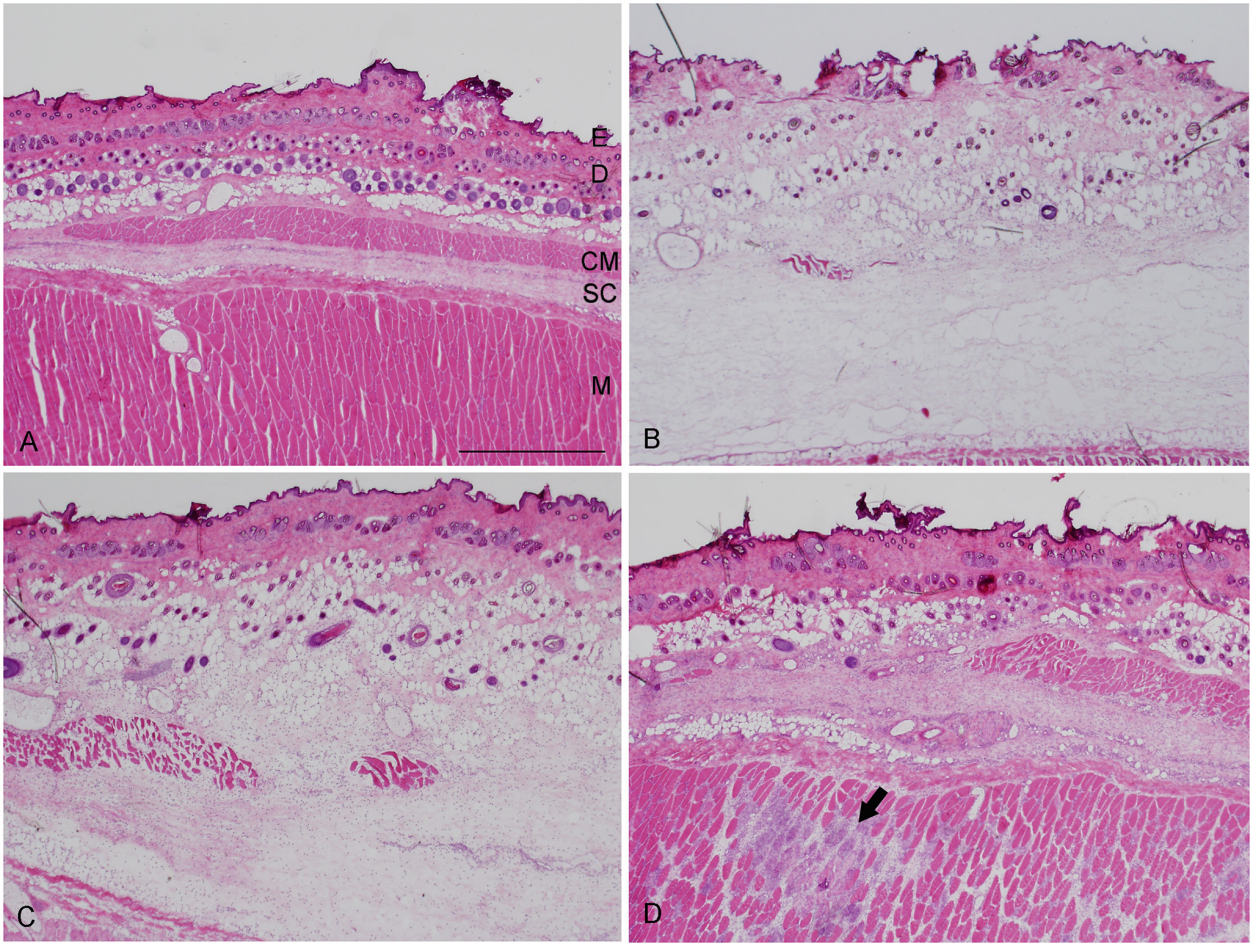
Hematoxylin and eosin stained sections of the abdominal wall. A typical example of each group is shown. a: sham group. The epidermis (E) is very thin. The skin and subcutaneous connective tissue are bordered by cutaneous muscle (CM). D; dermis, SC; subcutaneous connective tissue, M; muscle layer. b: At 12 hours after compression, the skin and subcutaneous connective tissue are markedly thickened. c: At 1 day after compression, the skin and subcutaneous connective tissue are still thickened. d: At 3 days after compression, thickness of skin and subcutaneous connective tissue are decreased. The arrow shows a necrotic area observed at the muscle layer, and many infiltrating cells are existing in this area. Scale bar = 1 mm.

The abdominal wall of rats was composed of the epidermis, dermis, skin muscle, subcutaneous tissue, and muscle layer [16]. The abdominal wall of the sham group was the same as for a normal skin structure (Fig 1a). At 12 hours, the dermis and the subcutaneous tissue were increased in thickness, and infiltrating cells were aggregated (Fig 1b). At 1 day, the dermis and the subcutaneous tissue were thinner than those of 12 hours, but edema was still observed (Fig 1c). Infiltrating cells were significantly increased in the dermis and subcutaneous tissue. At 3 days, edema was reduced, and fibroblast-like cells were accumulated in the subcutaneous tissue (Fig 1d).

### Microarray

GeneChip Rat Genome 230 2.0 Array revealed that numerous genes were up-regulated, e.g., 4607 genes at 12 hours, 4454 genes at 1 day, and 2368 genes at 3 days. Markedly up-regulated genes were described in Table in S1 File. A part of these up-regulated genes were associated with apoptosis, inflammation, oxidative stress, proteolysis, and hypoxia (Table A in S2 File). At 1 and 3 days, genes associated with proteolysis were markedly increased (Tables B, C in S2 File).

Alterations of inflammatory cytokines were represented in Table 2. GM-CSF, IL-1β, IL1Ra, IL-6, IL-10, IL-11, LT-β, MMP-3, TIMP-1, and TNF-α were markedly increased at 12 hours, and then decreased gradually to the level of the sham group at 1 and 3 days. IFN-γ, TGF-β2, and TGF-β3 were most increased at 1 day, and decreased to the level of the sham group at 3 days.

**Table 2:** A list of Taqman Assay kits used for Real-time PCR.

Abbreviation	Gene Name	Probe ID	12h		1d		3d	
			n = 1		n = 1		n = 1	
GM-CSF	granulocyte-macrophage colony stimulating factor	1371227_at	8.25	↑	1.91		0.61	
IFN-γ	interferon gamma	1370790_at	0.60		25.00	↑	3.71	↑
IL-1α	interleukin 1 alpha	1368592_at	1.96		0.72		1.42	
IL-1β	interleukin 1 beta	1398256_at	16.95	↑	0.24	↓	1.23	
IL1Ra	interleukin 1 receptor antagonist gene	1387835_at	8.31	↑	0.13	↓	0.98	
IL-2	interleukin 2	1369596_at	1.08		1.22		0.60	
IL-4	interleukin 4	1371128_at	1.27		0.78		0.49	↓
IL-6	interleukin 6 (interferon, beta 2)	1369191_at	82.42	↑	0.01	↓	0.74	
IL-10	interleukin 10	1387711_at	38.32	↑	0.25	↓	0.16	↓
IL-11	interleukin 11	1369534_at	6.08	↑	4.50	↑	1.07	
IL-12	interleukin 12	1369315_at	1.11		0.59		2.85	↑
LT-α	lymphotoxin alpha (TNF superfamily, member 1)	1368722_at	0.74		0.53		0.78	
LT-β	lymphotoxin beta (TNF superfamily, member 3)	1379499_at	15.21	↑	0.32	↓	1.24	
MMP-3	matrix metalloproteinase 3	1368657_at	22.18	↑	0.06	↓	0.61	
TGF-β1	transforming growth factor, beta 1	1370082_at	1.10		0.71		0.49	↓
TGF-β2	transforming growth factor, beta 2	1387172_a_at	0.69		2.72	↑	1.37	
TGF-β3	transforming growth factor, beta 3	1367859_at	1.11		2.13	↑	1.69	
TIMP-1	tissue inhibitor of metalloproteinase 1	1367712_at	4.83	↑	0.36	↓	0.40	↓
TIMP-2	TIMP metallopeptidase inhibitor 2	1386940_at	0.79		1.71		0.97	
TNF-α	tumor necrosis factor, alpha (cachetin)	1387691_at	6.05	↑	0.99		1.18	

Relative expression is calculated as the ratio of expression levels in compression group/sham group. Up-pointing arrows: up-regulated over twice, down-pointing arrows: down-regulated less than half.

Receptors of inflammatory cytokines such as GM-CSF, IL-1, IL-6, and TNF were slightly up-regulated (Table in S3 File). On the other hand, receptors for anti-inflammatory cytokines such as IL-2, IL-10, IL-11, and TGF-β were not changed (Table in S3 File).

### Real-Time PCR

**Fig 2:**
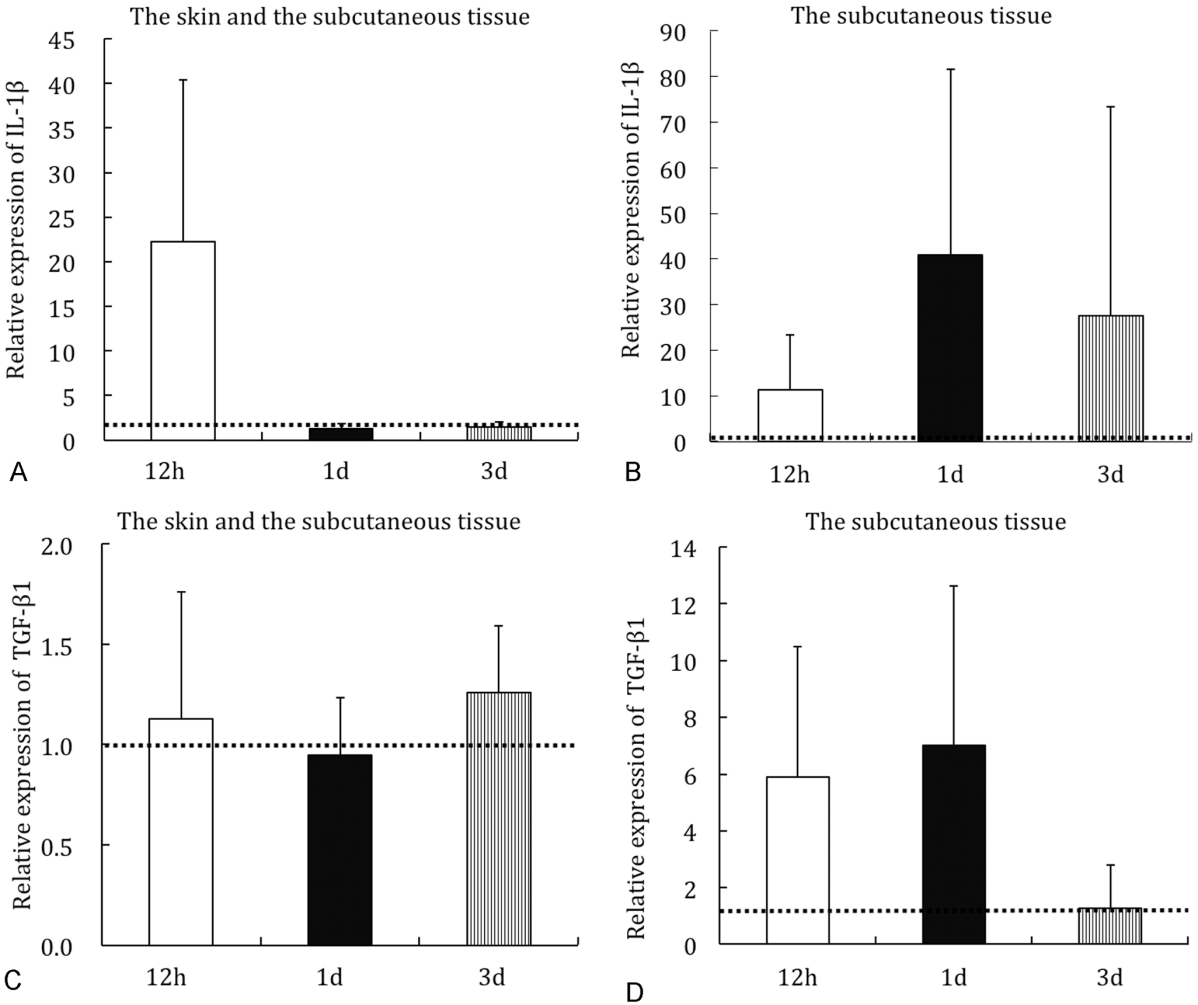
Relative mRNA expression level by Real-Time PCR. Part of the data of Table 2 were represented by a bar graph. IL-1β in the skin and the subcutaneous tissue (a), IL-1β in the subcutaneous tissue (b). TGF-β1 in the skin and the subcutaneous tissue (c), TGF-β1 in the subcutaneous tissue (d). The dotted line indicates the expression level in the sham group.

Some patterns of mRNA expression were revealed (Fig 2). Most of pro-inflammatory cytokines were up-regulated at 12 hours, and gradually decreased (Fig 2a and 2d). Expression of several cytokines remained high (Fig 2b). No changes were observed in some cytokines (Fig 2c).

Analysis of the skin and the subcutaneous tissue showed that GM-CSF, IL-1β, IL1Ra, IL-6, IL-10, and TNF-α were markedly increased at 12 hours, and gradually decreased to the level of the sham group at 1 and 3 days (Table 3). MMP-3 and TIMP-1 were most increased at 1 day, and decreased to the level of the sham group at 3 days.

**Table 3:** Results of Real-Time PCR analysis for inflammatory gene.

Abbreviation	the skin and the subcutaneous tissue								
	12h			1d			3d		
	n = 4			n = 4			n = 4		
GM-CSF	7.85	(2.93~17.41)	↑*	1.71	(1.00~3.49)		0.60	(0.34~1.02)	
IFN-γ	2.06	(0.76~3.84)	↑	2.26	(0.79~5.70)	↑	4.93	(1.96~8.01)	↑*
IL-1α	1.97	(0.86~3.75)		0.34	(0.17~0.44)	↓*	0.33	(0.25~0.47)	↓*
IL-1β	22.24	(4.88~41.34)	↑*	1.28	(0.65~1.80)		1.45	(0.88~2.16)	
IL-1Ra	3.98	(2.04~6.88)	↑*	0.73	(0.59~1.03)		0.54	(0.44~0.59)	
IL-2	0.64	(0.16~1.34)		0.40	(0.11~0.69)	↓	0.17	(0.08~0.28)	↓
IL-6	38.78	(26.26~66.03)	↑*	10.06	(6.85~12.76)	↑*	2.52	(1.12~4.53)	↑
IL-10	4.36	(2.23~5.59)	↑*	3.95	(2.46~6.29)	↑*	1.07	(0.71~1.63)	
MMP-3	9.01	(3.36~16.10)	↑*	12.54	(5.70~19.05)	↑*	2.61	(1.82~3.42)	↑*
TGF-β1	1.13	(0.56~1.86)		0.59	(0.59~1.19)		1.26	(1.03~1.74)	
TIMP-1	2.93	(2.00~4.02)	↑*	3.35	(2.07~4.14)	↑*	1.54	(1.27~1.68)	*
TNF-α	17.96	(3.07~39.39)	↑*	1.36	(0.90~1.64)		0.77	(0.59~1.16)	

RNAs from the skin and the subcutaneous tissue were analyzed. Values were described as fold changes (minimum value ~ maximum value). Up-pointing arrows: up-regulated over twice, down-pointing arrows: down-regulated less than half.

* p<0.05.

In addition, the subcutaneous tissue alone contained high level of cytokines (Table 4). GM-CSF was most increased at 12 hours, and decreased gradually at 1 and 3 days. IFN-γ, IL1Ra, MMP-3, and TGF-β1 were most increased at 1 day, and decreased after 3 days. On the other hand, IL-1α, IL-1β, IL-6, TIMP-1, and TNF-α were most increased at 1 day, remained highly expressed after 3 days.

**Table 4:** Results of Real-Time PCR analysis for inflammatory gene.

Abbreviation	the subcutaneous tissue								
	12h			1d			3d		
	n = 3			n = 3			n = 3		
GM-CSF	53.76	(13.32~119.96)	↑	7.52	(3.25~12.46)	↑	0.16	(0.04~0.40)	↓
IFN-γ	5.33	(1.43~12.60)	↑	7.87	(4.02~12.68)	↑	0.64	(0.09~1.81)	
IL-1α	5.72	(0.75~12.22)	↑	11.11	(2.90~25.89)	↑	6.33	(0.25~18.16)	↑
IL-1β	11.27	(3.31~25.04)	↑	40.87	(17.29~87.90)	↑	27.47	(0.92~80.32)	↑
IL-1Ra	3.67	(1.95~6.17)	↑	6.59	(3.62~12.24)	↑	1.19	(0.96~1.53)	
IL-2	1.31	(0.34~1.95)		0.61	(0.53~1.10)		0.49	(0.39~0.60)	↓
IL-6	2.66	(1.91~3.07)	↑	9.82	(2.96~16.46)	↑	6.31	(3.12~11.94)	↑
IL-10	0.64	(0.11~1.07)		0.42	(0.17~0.66)	↓	2.04	(1.13~2.58)	↑
MMP-3	4.38	(1.30~8.96)	↑	34	(30.34~36.25)	↑	1.58	(1.00~1.91)	
TGF-β1	5.9	(0.63~8.60)	↑	7.01	(0.85~11.80)	↑	1.27	(0.37~3.02)	
TIMP-1	3.28	(3.11~3.57)	↑	3.27	(2.57~4.38)	↑	3.13	(2.01~4.24)	↑
TNF-α	0.68	(0.48~0.99)		2.13	(1.04~4.10)	↑	3.31	(2.05~3.95)	↑

RNAs from the subcutaneous tissue were analyzed. Values were described as fold changes (minimum value ~ maximum value). Up-pointing arrows: up-regulated over twice, down-pointing arrows: down-regulated less than half.

### ELISA

The ELISA results showed a slight increase in IL-1β and IL-6 concentrations (Table 5). At 12 hours after compression, IL-1β mRNA was dramatically increased, but the protein concentration was increased to a much lesser extent. In addition, at 1 day and 3 days after compression, IL-1β levels were lower than in the sham group. On the other hand, at 12 h and 1 day after compression, IL-6 levels were higher in the compression group than in the sham group. Contrary to the results from the microarray and RT-PCR experiments, ELISA showed that IFN-γ, IL-1α, IL-10, and TNF-α were insignificantly changed compared to IL-1β and IL-6.

**Table 5:** Results of the ELISAs for detection of inflammatory cytokines.

Abbreviation	the skin and the subcutaneous tissue					
	12h		1d		3d	
IFN-γ	0.84	(0.57~1.14)	1.44	(0.76~2.02)	0.3	(0.13~0.51)
IL-1α	0.63	(0.56~0.75)	0.37	(0.17~0.62)	0.52	(0.37~0.75)
IL-1β	1.61	(1.02~2.13)	0.75	(0.34~1.64)	0.52	(0.35~0.63)
IL-6	1.99	(0.66~4.02)	2.49	(1.42~4.03)	0.43	(0.14~0.80)
IL-10	0.37	(0.09~0.55)	1.32	(0.91~1.71)	0.52	(0.34~0.76)
TNF-α	0.28	(0.20~0.41)	1.12	(0.72~1.48)	0.67	(0.61~0.79)

Proteins extracted from the skin and subcutaneous tissues were analyzed. Values were described as fold changes (minimum value ~ maximum value).

## Discussion

### Gene expressions associated with an inflammatory response

In this study, mRNA levels of several cytokines were increased, and many of these cytokines have been reported to be increased after continuous ischemia [17], [18] or re-perfusion before[19]. Among these cytokines, IL-1β, IL-6, and TNF-α are well known to cause local inflammation [14], [20], so the significant increments of these genes in the compressed area would be associated with inflammation.

In addition, an increment of GM-CSF, IFN-γ, IL-10, MMP-3, and TIMP-1 was shown in this study. However, the roles of these cytokines in the generation of pressure ulcers were not reported. GM-CSF is known to activate different kinds of cells, such as macrophages, neutrophils, monocytes, mast cells, and eosinophils [21], and induces the aggregation of neutrophils at the inflammation site [22]. Microscopic observation revealed that infiltrating cells were increased at 12 hours and 1 day. Therefore, GM-CSF seems to contribute to infiltration and activation of inflammatory cells in the early phase. IFN-γ has a role in activation of macrophages, and this cytokine increased at different time point from each analysis. So, IFN-γ must be checked precisely when the peak of expression occurs. MMP-3 is a key enzyme involved in the breakdown of the extracellular matrix [23]. High expression of MMP-3 may cause breakdown in the compressed area, and might cause the loosening of the skin and the subcutaneous tissue. As a result of this, the skin and the subcutaneous tissue could be thickened. The role of IL-10 and TIMP-1 is inhibition of inflammation [14], [23–25]. The up-regulated levels of these genes were lower than that of inflammatory cytokines such as IL-1β, IL-6, and TNF-α. So, edema and redness occurred despite IL-10 and TIMP-1 up-regulation.

The mRNA of inflammatory cytokines was markedly increased, however the increments of those proteins were limited. In this study, we observed the alteration after a single compression. However, severe damage occurred after repeated compression [26]. Tissue only reversibly healed after two or three repeated compressions. So, the influence of inflammatory cytokines should be further investigated.

### Changes in the subcutaneous tissue

In the subcutaneous tissue, remarkable edema and a number of infiltrating cells were observed by microscopy. Real-Time PCR revealed that several inflammatory cytokines were up-regulated. In addition, some cytokines kept at high level at 3 days. These findings suggest that the subcutaneous tissue has important role in the generation of pressure ulcers.

The subcutaneous tissue mostly consists of connective tissue and a small number of cells. Because of the looseness, connective tissue and the cells in this layer could be affected easily by external force [16], [27]. Continuous compressions transduce mechanical stress to subcutaneous tissue, and cells in this layer would express several cytokines. Light microscopy showed that infiltrating cells were aggregated in acute phase, and numerous fibroblast-like cells were observed after 3 days. These cells in the subcutaneous tissue may associate with the production of the inflammatory cytokines. Stojadinovic *et al*. [28] showed that the subepidermal separation affected to the deep tissue injury and our results also showed the importance of deep tissue alteration.

### Responsible genes for pressure ulcers formation

In the subcutaneous tissue, some genes were up-regulated temporarily, and expression of other gene was kept at a high level. Temporally up-regulated genes, such as IL1Ra, MMP-3, and TGF-β1, could work at the early phase. On the other hand, IL-1α, IL-1β, IL-6, TIMP-1, and TNF-α were highly up-regulated even after 3 days. Chen *et al*. showed that IL-1α, IL-1β, IL-6, and TNF-α have an important role in the wound healing process [29]. IL-1α, IL-1β, IL-6, and TNF-α trigger or enhance inflammation [14], and these pro-inflammatory cytokines were increased markedly more than IL1Ra and TGF-β1, which inhibit inflammation. Anti-inflammatory cytokines were up-regulated, but the increment was much lower than for pro-inflammatory cytokines. Anti- inflammatory cytokines such as IL-10 and IL1Ra have the possibility to suppress the inflammatory stimuli [25]. IL1Ra and TGF-β1 seem to be insufficient to suppress inflammation. Much more inflammatory cytokines perhaps induce enhanced inflammation [14]. The ELISA data showed a slight increase in IL-1β and IL-6, while IL-10 was decreased. This suggests an imbalance of inflammatory cytokines and anti-inflammatory cytokines. IL-1β and IL-6 were increased in preference of other cytokines, which may indicate a superior role of IL-1β and IL-6 in the formation of pressure ulcers.

MMP-3 decreased at 3 days, and its inhibitor TIMP-1 was highly expressed. TIMP-1 binds MMPs to inhibit the activation of MMP and promotes the cells to proliferate [23]. So, enough TIMP-1 may be expressed to contribute the morphological repair. A previous study showed the importance of anti-inflammatory cytokines in the wound healing process, but not in pressure ulcers formation. Many studies investigating pressure ulcers compress the skin at clinically unreal pressure. A weak pressure only could enhance the expression of anti-inflammatory cytokines, and strong compression might cause the restriction of those genes.

The microarray also revealed that some receptors of inflammatory cytokines (e.g. GM-CSF, IL-1, IL-6, and TNF-α) were up-regulated, and receptors of anti-inflammatory cytokines were not changed (Table in S3 File). These data suggest that an inflammatory signal was activated, and that especially GM-CSF, IL-1, IL-6, and TNF-α contributed to the generation of the pressure ulcers.

In conclusion, the microarray showed that the single compression could induce the up-regulation of numerous genes. Lasting up-regulation of inflammatory cytokines occurred mainly in the subcutaneous tissue. Most of the up-regulated cytokines could work as activators of inflammatory cells, and those cytokines may be associated with local tissue damage. However, the role of inflammatory cytokines was not definitive. Our data showed the up-regulation of mRNA of inflammatory cytokines, but the elevation of those proteins could not be confirmed. In addition, we showed the up-regulation of inflammatory cytokines in healthy rats, but pressure ulcers are often generated in compromised patients. The role of those cytokines in the formation of human pressure ulcers must be further investigated. We focused on inflammatory cytokines, but other altered genes also have a possible contribution to the pressure ulcer formation. Our microarray data are very useful to gain a further understanding of pressure ulcers, and continued investigation could lead to the development of preventive or therapeutic methods.

## Supporting Information

S1 File Genes up-regulated in the skin and subcutaneous tissue revealed by microarray analysis. The values are at 12 hours (Table A), at 1 day (Table B), and 3 days (Table C) after compression. Two hundred kinds of genes were sequentially arranged in descending order of expression level. EST means Expressed Sequence Tag, and it is a short sequence of a transcribed spliced nucleotide sequence.
(DOCX)

S2 File Classification of up-regulated genes. The values are at 12 hours (Table A), at 1 day (Table B), and 3 days (Table C) after compression. Biological process classified by gene ontology (obtained from NetAffx Analysis Center) are represented at left column, and the number of genes which increased over twice.
(DOCX)

S3 File Results of Microarray analysis with receptors to inflammatory cytokine. RNAs from the skin and the subcutaneous tissue analyzed. Up-pointing arrows: up-regulated over twice, down-pointing arrows: down-regulated less than half.
(DOCX)
