# Supplementary material for: Analysis of Gene Expression in Experimental Pressure Ulcers in the Rat with Special Reference to Inflammatory Cytokines
Source: PLoS One. 2015 Jul 15;10(7):e0132622. doi: 10.1371/journal.pone.0132622 (PMC4503587; doi:10.1371/journal.pone.0132622)
Supplement: S1 File — The values are at 12 hours (Table A), at 1 day (Table B), and 3 days (Table C) after compression. Two hundred kinds of genes were sequentially arranged in descending order of expression level. EST means Expressed Sequence Tag, and it is a short sequence of a transcribed spliced nucleotide sequence. (DOCX) [file pone.0132622.s001.docx]

Table A. Genes up-regulated in the skin and subcutaneous tissue revealed by microarray analysis at 12 hours after compression.

|  | Gene Symbol | Gene Name | Probe Set ID | Fold Change |
| --- | --- | --- | --- | --- |
|  |  |  |  | compression/sham |
| 1 | Cxcl6 | chemokine (C-X-C motif) ligand 6 | 1387648_at | 328.39 |
| 2 | Cxcl1 | chemokine (C-X-C motif) ligand 1 | 1387316_at | 148.11 |
| 3 | Pglyrp1 | peptidoglycan recognition protein 1 | 1387422_at | 107.85 |
| 4 | Mmp9 | matrix metallopeptidase 9 | 1398275_at | 99.16 |
| 5 | Cxcl2 | chemokine (C-X-C motif) ligand 2 | 1368760_at | 99.06 |
| 6 | RGD1561734 | similar to KIAA1913 | 1378348_at | 90.00 |
| 7 | RGD1563047 | similar to von Ebner minor salivary gland protein | 1381964_at | 89.61 |
| 8 | Il6 | interleukin 6 | 1369191_at | 82.42 |
| 9 | EST | EST | 1398576_at | 80.91 |
| 10 | Mmp9 | matrix metallopeptidase 9 | 1369166_at | 70.08 |
| 11 | EST | EST | 1385861_x_at | 69.67 |
| 12 | EST | EST | 1381599_at | 68.25 |
| 13 | Klhl21 | kelch-like 21 (Drosophila) | 1396277_at | 66.00 |
| 14 | Prok2 | prokineticin 2 | 1369622_at | 60.34 |
| 15 | Csf3r | colony stimulating factor 3 receptor (granulocyte) | 1386009_at | 54.33 |
| 16 | Fosl1 | fos-like antigen 1 | 1368489_at | 52.81 |
| 17 | Cxcl3 | chemokine (C-X-C motif) ligand 3 | 1370634_x_at | 51.54 |
| 18 | Serpine1 | serine (or cysteine) peptidase inhibitor, clade E, member 1 | 1368519_at | 50.23 |
| 19 | Ccl2 | chemokine (C-C motif) ligand 2 | 1367973_at | 49.31 |
| 20 | Cyct / Pde11a | cytochrome c, testis / phosphodiesterase 11A | 1387534_at | 49.00 |
| 21 | Tnfrsf1b | tumor necrosis factor receptor superfamily, member 1b | 1392731_at | 48.42 |
| 22 | Cxcl3 | chemokine (C-X-C motif) ligand 3 | 1370633_at | 47.29 |
| 23 | Il8rb | interleukin 8 receptor, beta | 1369697_at | 45.47 |
| 24 | EST | EST | 1381498_at | 45.40 |
| 25 | Tsx | testis specific X-linked gene | 1368736_at | 44.29 |
| 26 | Fga | fibrinogen, alpha polypeptide | 1371258_at | 43.05 |
| 27 | Fga | fibrinogen, alpha polypeptide | 1370992_a_at | 42.40 |
| 28 | Mmp8 | matrix metallopeptidase 8 | 1387735_at | 40.89 |
| 29 | Slc24a2 | solute carrier family 24 (sodium/potassium/calcium exchanger), member 2 | 1388000_at | 39.00 |
| 30 | Fcnb | ficolin B | 1387378_at | 38.94 |
| 31 | Il10 | interleukin 10 | 1387711_at | 38.32 |
| 32 | EST | EST | 1376375_at | 37.33 |
| 33 | EST | EST | 1379679_at | 34.94 |
| 34 | Cxcl3 | chemokine (C-X-C motif) ligand 3 | 1388032_a_at | 34.19 |
| 35 | Nos2 | nitric oxide synthase 2, inducible, macrophage | 1387667_at | 32.93 |
| 36 | EST | EST | 1375403_at | 32.69 |
| 37 | EST | EST | 1394810_at | 32.50 |
| 38 | Camp | cathelicidin antimicrobial peptide | 1393603_at | 32.46 |
| 39 | Sell | selectin, lymphocyte | 1369801_at | 32.19 |
| 40 | Ccl4 | chemokine (C-C motif) ligand 4 | 1370832_at | 31.53 |
| 41 | Thsd7b | thrombospondin, type I, domain containing 7B | 1385744_at | 31.25 |
| 42 | Sprr3 | small proline-rich protein 3 | 1376959_at | 30.88 |
| 43 | Hp | haptoglobin | 1370148_at | 30.64 |
| 44 | Slfn3 | schlafen 3 | 1387134_at | 30.35 |
| 45 | EST | EST | 1394976_at | 29.38 |
| 46 | Kars | lysyl-tRNA synthetase | 1398106_at | 29.20 |
| 47 | RGD1564357 | RGD1564357 | 1381082_at | 29.07 |
| 48 | EST | EST | 1377942_at | 28.75 |
| 49 | EST | EST | 1384165_at | 27.40 |
| 50 | EST | EST | 1376742_at | 27.38 |
| 51 | EST | EST | 1389823_at | 26.78 |
| 52 | Slc13a1 | solute carrier family 13 (sodium/sulfate symporters), member 1 | 1370468_at | 26.75 |
| 53 | EST | EST | 1380885_at | 25.96 |
| 54 | Rfxdc2 | regulatory factor X domain containing 2 homolog (human) | 1379871_at | 25.87 |
| 55 | EST | EST | 1382989_at | 25.67 |
| 56 | EST | EST | 1397658_at | 25.60 |
| 57 | RGD1562846 | similar to Docking protein 5 (Downstream of tyrosine kinase 5) (Protein dok-5) | 1396206_at | 25.54 |
| 58 | PVR | poliovirus receptor | 1370177_at | 25.38 |
| 59 | EST | EST | 1396889_at | 25.21 |
| 60 | Irx1 | Iroquois related homeobox 1 (Drosophila) | 1377177_at | 25.18 |
| 61 | Nfe2 | nuclear factor, erythroid derived 2 | 1375040_at | 25.04 |
| 62 | EST | EST | 1391989_at | 24.90 |
| 63 | Pgs1 | Phosphatidylglycerophosphate synthase 1 | 1391328_at | 24.81 |
| 64 | EST | EST | 1381121_at | 24.69 |
| 65 | RGD1307493 | similar to membrane protein expressed in epithelial-like lung adenocarcinoma | 1389992_at | 24.65 |
| 66 | EST | EST | 1390724_at | 24.63 |
| 67 | EST | EST | 1396868_at | 24.57 |
| 68 | EST | EST | 1380338_at | 24.46 |
| 69 | Dopey2 | Dopey family member 2 | 1392458_at | 24.14 |
| 70 | EST | EST | 1395193_at | 23.93 |
| 71 | EST | EST | 1397019_at | 23.83 |
| 72 | Ccl7 | chemokine (C-C motif) ligand 7 | 1379935_at | 23.55 |
| 73 | Akr7a3 | aldo-keto reductase family 7, member A3 (aflatoxin aldehyde reductase) | 1368121_at | 23.34 |
| 74 | EST | EST | 1381735_at | 23.20 |
| 75 | Tfpi2 | tissue factor pathway inhibitor 2 | 1377340_at | 23.11 |
| 76 | Clec7a | C-type lectin domain family 7, member a | 1382692_at | 23.09 |
| 77 | Prl3a1 | Prolactin family 3, subfamily a, member 1 | 1370441_at | 22.83 |
| 78 | EST | EST | 1383449_at | 22.50 |
| 79 | EST | EST | 1377561_at | 22.29 |
| 80 | RGD1563946 | similar to mKIAA1623 protein | 1396653_at | 22.21 |
| 81 | Mmp3 | matrix metallopeptidase 3 | 1368657_at | 22.18 |
| 82 | EST | EST | 1377529_at | 22.17 |
| 83 | Ppp1r3d | Protein phosphatase 1, regulatory subunit 3D | 1378270_at | 22.14 |
| 84 | EST | EST | 1397626_at | 22.07 |
| 85 | RGD1309784 | Similar to ribosomal protein L24-like; 60S ribosomal protein L30 isolog; my024 protein; homolog of yeast ribosomal like protein 24 | 1395449_at | 22.05 |
| 86 | LOC687992 | similar to Serum amyloid A-3 protein precursor | 1392647_at | 21.96 |
| 87 | EST | EST | 1397661_at | 21.73 |
| 88 | Cfh / Cfhl1 | complement component factor H / complement component factor h-like 1 | 1382678_at | 21.64 |
| 89 | EST | EST | 1381965_at | 21.60 |
| 90 | EST | EST | 1398189_at | 21.35 |
| 91 | EST | EST | 1375139_at | 21.35 |
| 92 | RGD1309707 | similar to RIKEN cDNA 4930431E10 | 1392802_at | 21.32 |
| 93 | EST | EST | 1395954_at | 21.25 |
| 94 | Serpine1 | serine (or cysteine) peptidase inhibitor, clade E, member 1 | 1392264_s_at | 21.18 |
| 95 | RGD1565350 | Similar to Shb protein | 1375810_at | 21.17 |
| 96 | EST | EST | 1376014_at | 21.00 |
| 97 | Dcbld2 | discoidin, CUB and LCCL domain containing 2 | 1387451_at | 20.95 |
| 98 | EST | EST | 1391227_at | 20.92 |
| 99 | Slc35f4 | solute carrier family 35, member F4 | 1392206_at | 20.63 |
| 100 | Pax1 | paired box gene 1 | 1391678_at | 20.60 |
| 101 | EST | EST | 1371097_at | 20.57 |
| 102 | Cep76 | Centrosomal protein 76 | 1381372_at | 20.50 |
| 103 | EST | EST | 1379497_at | 20.48 |
| 104 | EST | EST | 1396814_at | 20.46 |
| 105 | Andpro | androgen regulated 20 kDa protein | 1367558_x_at | 20.25 |
| 106 | EST | EST | 1398092_at | 20.23 |
| 107 | EST | EST | 1378671_at | 20.22 |
| 108 | EST | EST | 1396927_at | 20.14 |
| 109 | EST | EST | 1390726_at | 19.96 |
| 110 | EST | EST | 1384666_at | 19.67 |
| 111 | EST | EST | 1380925_at | 19.65 |
| 112 | EST | EST | 1397971_at | 19.50 |
| 113 | EST | EST | 1396781_at | 19.45 |
| 114 | Il24 | interleukin 24 | 1368210_at | 19.44 |
| 115 | EST | EST | 1394839_at | 19.42 |
| 116 | Napsa | napsin A aspartic peptidase | 1368521_at | 19.38 |
| 117 | EST | EST | 1397968_at | 19.35 |
| 118 | Lilrb4 / LOC499078 | leukocyte immunoglobulin-like receptor, subfamily B, member 4 / similar to GP49B1 | 1375917_at | 19.28 |
| 119 | LOC290876 | similar to RIKEN cDNA 1700029H14 | 1385348_at | 19.17 |
| 120 | Neto1 | neuropilin (NRP) and tolloid (TLL)-like 1 | 1385492_at | 18.90 |
| 121 | Sstr2 | somatostatin receptor 2 | 1368782_at | 18.88 |
| 122 | EST | EST | 1394898_at | 18.86 |
| 123 | EST | EST | 1394805_at | 18.80 |
| 124 | Hmgb4 | high-mobility group box 4 | 1393986_at | 18.75 |
| 125 | Gldc | Glycine decarboxylase | 1379334_at | 18.69 |
| 126 | Hand2 | heart and neural crest derivatives expressed transcript 2 | 1369817_at | 18.65 |
| 127 | EST | EST | 1384513_at | 18.60 |
| 128 | Rdh5 | retinol dehydrogenase 5 | 1393865_at | 18.59 |
| 129 | Apbb2 | amyloid beta (A4) precursor protein-binding, family B, member 2 | 1397071_at | 18.56 |
| 130 | EST | EST | 1378011_at | 18.40 |
| 131 | LOC286911 | cationic trypsinogen | 1388186_at | 18.31 |
| 132 | Vcan | versican | 1371233_at | 18.28 |
| 133 | EST | EST | 1396663_at | 18.25 |
| 134 | EST | EST | 1376526_at | 18.24 |
| 135 | Prkag2 | Protein kinase, AMP-activated, gamma 2 non-catalytic subunit | 1375835_at | 18.18 |
| 136 | EST | EST | 1397318_at | 18.18 |
| 137 | Foxa3 | forkhead box A3 | 1387506_at | 18.14 |
| 138 | EST | EST | 1384600_at | 18.08 |
| 139 | EST | EST | 1384738_at | 18.08 |
| 140 | EST | EST | 1396932_at | 18.00 |
| 141 | Ccl3 | chemokine (C-C motif) ligand 3 | 1369815_at | 17.86 |
| 142 | Il1rl1 | interleukin 1 receptor-like 1 | 1387273_at | 17.83 |
| 143 | EST | EST | 1396397_at | 17.82 |
| 144 | EST | EST | 1386168_at | 17.79 |
| 145 | EST | EST | 1389887_at | 17.78 |
| 146 | EST | EST | 1393501_at | 17.78 |
| 147 | EST | EST | 1395569_at | 17.78 |
| 148 | EST | EST | 1391505_x_at | 17.75 |
| 149 | EST | EST | 1391877_at | 17.65 |
| 150 | EST | EST | 1380540_at | 17.55 |
| 151 | EST | EST | 1395222_at | 17.53 |
| 152 | EST | EST | 1397069_at | 17.45 |
| 153 | EST | EST | 1398186_at | 17.43 |
| 154 | RGD1564972 | RGD1564972 | 1380291_at | 17.38 |
| 155 | EST | EST | 1395278_at | 17.36 |
| 156 | EST | EST | 1396753_at | 17.24 |
| 157 | EST | EST | 1382742_at | 17.06 |
| 158 | Il1b | interleukin 1 beta | 1398256_at | 16.95 |
| 159 | EST | EST | 1391493_at | 16.92 |
| 160 | EST | EST | 1396686_at | 16.80 |
| 161 | LOC685048 / LOC685111 / RGD1559588 / Vom2r61 | similar to paired immunoglobin-like type 2 receptor beta / similar to paired immunoglobin-like type 2 receptor beta / similar to cell surface receptor FDFACT / vomeronasal 2 receptor, 61 | 1385047_x_at | 16.75 |
| 162 | LOC56764 | dnaj-like protein | 1368663_at | 16.71 |
| 163 | Rgs1 | regulator of G-protein signaling 1 | 1398540_at | 16.65 |
| 164 | Slc2a3 | solute carrier family 2 (facilitated glucose transporter), member 3 | 1387707_at | 16.62 |
| 165 | EST | EST | 1392401_s_at | 16.60 |
| 166 | EST | EST | 1397560_at | 16.52 |
| 167 | Selp | selectin, platelet | 1391946_at | 16.50 |
| 168 | EST | EST | 1397062_at | 16.47 |
| 169 | Neurog3 | neurogenin 3 | 1369853_at | 16.41 |
| 170 | EST | EST | 1380974_at | 16.41 |
| 171 | EST | EST | 1376186_at | 16.34 |
| 172 | EST | EST | 1379088_x_at | 16.33 |
| 173 | EST | EST | 1375499_at | 16.28 |
| 174 | EST | EST | 1375617_at | 16.24 |
| 175 | Kbtbd7 | Kelch repeat and BTB (POZ) domain containing 7 | 1377930_at | 16.20 |
| 176 | EST | EST | 1396709_at | 16.15 |
| 177 | EST | EST | 1394538_at | 16.13 |
| 178 | EST | EST | 1392375_at | 16.03 |
| 179 | Gsta2 / Gsta3 / Yc2 | glutathione-S-transferase, alpha type2 / glutathione S-transferase A3 / glutathione S-transferase Yc2 subunit | 1371089_at | 16.00 |
| 180 | EST | EST | 1395005_at | 16.00 |
| 181 | LOC685756 | Hypothetical protein LOC685756 | 1378553_at | 15.91 |
| 182 | Slco1c1 | solute carrier organic anion transporter family, member 1c1 | 1370205_at | 15.83 |
| 183 | EST | EST | 1395874_at | 15.78 |
| 184 | Arid1b / LOC497729 | AT rich interactive domain 1B (Swi1 like) / hypothetical gene supported by NM_172157 | 1379856_at | 15.74 |
| 185 | Cdh1 | cadherin 1 | 1386446_at | 15.73 |
| 186 | EST | EST | 1396212_at | 15.65 |
| 187 | Prnd | prion protein dublet | 1384924_at | 15.56 |
| 188 | Cntn5 | contactin 5 | 1369366_at | 15.55 |
| 189 | EST | EST | 1378867_at | 15.47 |
| 190 | Smad6 | MAD homolog 6 (Drosophila) | 1377467_at | 15.23 |
| 191 | Ltb | lymphotoxin B | 1379499_at | 15.21 |
| 192 | Sfrs2ip | Splicing factor, arginine/serine-rich 2, interacting protein | 1383043_at | 15.18 |
| 193 | EST | EST | 1377175_at | 15.11 |
| 194 | Bst1 | bone marrow stromal cell antigen 1 | 1369294_at | 15.10 |
| 195 | EST | EST | 1374261_at | 15.08 |
| 196 | RGD1562704 | RGD1562704 | 1392028_at | 15.05 |
| 197 | EST | EST | 1377195_at | 15.04 |
| 198 | EST | EST | 1392741_at | 14.94 |
| 199 | EST | EST | 1396681_at | 14.94 |
| 200 | EST | EST | 1375797_at | 14.93 |

Table B. Genes up-regulated in the skin and subcutaneous tissue revealed by microarray analysis at 1 day after compression.

|  | Gene Symbol | Gene Name | Probe Set ID | Fold Change |
| --- | --- | --- | --- | --- |
|  |  |  |  | compression/sham |
| 1 | RT1-Bb | RT1 class II, locus Bb | 1371033_at | 211.24 |
| 2 | EST | EST | 1381337_at | 103.00 |
| 3 | Adh6a | alcohol dehydrogenase 6A (class V) | 1381756_at | 95.78 |
| 4 | EST | EST | 1382031_at | 88.00 |
| 5 | Amy1 / Amy2 | amylase 1, salivary / amylase 2, pancreatic | 1369502_a_at | 78.36 |
| 6 | EST | EST | 1398716_at | 76.33 |
| 7 | EST | EST | 1392809_at | 70.00 |
| 8 | Col11a1 | collagen, type XI, alpha 1 | 1384211_at | 67.62 |
| 9 | EST | EST | 1392127_at | 65.67 |
| 10 | RGD1562234 | similar to S100 calcium-binding protein, ventral prostate | 1373276_at | 64.96 |
| 11 | Abca8b | ATP-binding cassette, sub-family A (ABC1), member 8b | 1395644_at | 62.38 |
| 12 | EST | EST | 1391961_a_at | 55.03 |
| 13 | Col11a1 | collagen, type XI, alpha 1 | 1384931_at | 52.00 |
| 14 | EST | EST | 1381595_at | 51.50 |
| 15 | Col11a1 | collagen, type XI, alpha 1 | 1392915_at | 49.68 |
| 16 | Tore | trispanning orphan | 1369585_at | 48.86 |
| 17 | A2bp1 | ataxin 2 binding protein 1 | 1380516_at | 47.50 |
| 18 | EST | EST | 1393434_at | 47.00 |
| 19 | EST | EST | 1371910_at | 45.83 |
| 20 | Hmcn2 | hemicentin 2 | 1390236_at | 45.58 |
| 21 | EST | EST | 1395692_at | 45.13 |
| 22 | EST | EST | 1386430_at | 44.80 |
| 23 | EST | EST | 1385078_at | 44.67 |
| 24 | Pi16 | peptidase inhibitor 16 | 1380484_at | 42.75 |
| 25 | Prl8a4 | prolactin family 8, subfamily a, member 4 | 1386919_at | 42.00 |
| 26 | EST | EST | 1382808_at | 42.00 |
| 27 | EST | EST | 1384346_at | 41.80 |
| 28 | EST | EST | 1376752_at | 41.50 |
| 29 | EST | EST | 1395112_at | 40.58 |
| 30 | EST | EST | 1373478_at | 40.56 |
| 31 | Lgi1 | leucine-rich repeat LGI family, member 1 | 1386023_at | 40.52 |
| 32 | Ncor1 | Nuclear receptor co-repressor 1 | 1392175_at | 40.40 |
| 33 | Fbn2 | fibrillin 2 | 1370037_at | 39.30 |
| 34 | EST | EST | 1376375_at | 38.00 |
| 35 | Plcd4 | phospholipase C, delta 4 | 1387065_at | 38.00 |
| 36 | EST | EST | 1378603_at | 37.90 |
| 37 | EST | EST | 1393064_at | 37.41 |
| 38 | Mpped2 | metallophosphoesterase domain containing 2 | 1392077_at | 37.13 |
| 39 | EST | EST | 1390562_s_at | 37.13 |
| 40 | Zmynd17 | zinc finger, MYND domain containing 17 | 1389436_at | 36.85 |
| 41 | Trpv5 | transient receptor potential cation channel, subfamily V, member 5 | 1370644_at | 36.57 |
| 42 | EST | EST | 1377538_at | 36.50 |
| 43 | Slc23a3 | solute carrier family 23 (nucleobase transporters), member 3 | 1386454_at | 36.08 |
| 44 | EST | EST | 1397700_x_at | 36.00 |
| 45 | Mlana | melan-A | 1390596_at | 35.67 |
| 46 | Mak | male germ cell-associated kinase | 1387324_at | 34.86 |
| 47 | EST | EST | 1389284_at | 34.16 |
| 48 | Uts2r | urotensin 2 receptor | 1387304_at | 33.43 |
| 49 | EST | EST | 1384158_at | 33.00 |
| 50 | EST | EST | 1395904_at | 33.00 |
| 51 | Lepr | leptin receptor | 1370605_s_at | 32.82 |
| 52 | Lrrc19 | leucine rich repeat containing 19 | 1382840_at | 32.33 |
| 53 | EST | EST | 1394126_at | 31.33 |
| 54 | EST | EST | 1375690_at | 31.31 |
| 55 | EST | EST | 1396665_at | 30.80 |
| 56 | Itgb1bp1 | integrin beta 1 binding protein 1 | 1378448_at | 30.71 |
| 57 | Akr1d1 | aldo-keto reductase family 1, member D1 | 1392384_s_at | 30.40 |
| 58 | Hspa12b | Heat shock protein 12B | 1394442_at | 30.28 |
| 59 | Angptl1 | angiopoietin-like 1 | 1392832_at | 30.15 |
| 60 | Bai2 | brain-specific angiogenesis inhibitor 2 | 1378871_at | 29.84 |
| 61 | Tinag | tubulointerstitial nephritis antigen | 1392411_at | 29.60 |
| 62 | Mrap | melanocortin 2 receptor accessory protein | 1379491_at | 29.52 |
| 63 | Trpc5 | transient receptor potential cation channel, subfamily C, member 5 | 1369368_at | 29.45 |
| 64 | Pbsn | probasin | 1387191_at | 29.00 |
| 65 | RGD1564833 | similar to 9630058J23Rik protein | 1393264_at | 28.76 |
| 66 | EST | EST | 1388845_at | 28.53 |
| 67 | LOC682990 / LOC683021 / LOC685110 / LOC685347 / LOC685392 / LOC685459 / LOC685544 / RGD1565859 | hypothetical protein LOC682990 / hypothetical protein LOC683021 / hypothetical protein LOC685110 / hypothetical protein LOC685347 / hypothetical protein LOC685392 / hypothetical protein LOC685459 / hypothetical protein LOC685544 / RGD1565859 | 1394125_at | 28.35 |
| 68 | Hoxb8 | homeo box B8 | 1380142_at | 28.33 |
| 69 | EST | EST | 1374187_at | 28.30 |
| 70 | LOC689212 / LOC689220 / RGD1560483 | similar to Cystatin S precursor (LM protein) / similar to Cystatin S precursor (LM protein) / similar to Cystatin S precursor (LM protein) | 1381398_at | 28.25 |
| 71 | Cst12 | cystatin 12 | 1370404_at | 28.18 |
| 72 | EST | EST | 1393108_at | 28.08 |
| 73 | EST | EST | 1377368_at | 27.60 |
| 74 | Cyp2c13 | cytochrome P450 2c13 | 1370495_s_at | 27.25 |
| 75 | EST | EST | 1391500_at | 27.03 |
| 76 | EST | EST | 1392359_at | 27.00 |
| 77 | Akr1b7 | aldo-keto reductase family 1, member B7 | 1368569_at | 26.75 |
| 78 | Vipr2 | vasoactive intestinal peptide receptor 2 | 1387177_at | 26.59 |
| 79 | EST | EST | 1380351_at | 26.43 |
| 80 | EST | EST | 1392360_at | 26.33 |
| 81 | Gen1 | Gen homolog 1, endonuclease (Drosophila) | 1384102_at | 26.13 |
| 82 | EST | EST | 1397123_at | 25.75 |
| 83 | EST | EST | 1396316_at | 25.69 |
| 84 | EST | EST | 1385260_at | 25.14 |
| 85 | Cyp3a23/3a1 | cytochrome P450, family 3, subfamily a, polypeptide 23/polypeptide 1 | 1387118_at | 25.00 |
| 86 | Ifng | interferon gamma | 1370790_at | 25.00 |
| 87 | EST | EST | 1394900_at | 25.00 |
| 88 | Selenbp1 | selenium binding protein 1 | 1367673_at | 24.69 |
| 89 | LOC684878 / LOC691396 | similar to Zinc finger protein 551 (Zinc finger protein KOX23) / similar to Zinc finger protein 551 (Zinc finger protein KOX23) | 1390725_at | 24.62 |
| 90 | Cd5l | CD5 antigen-like | 1386843_at | 24.44 |
| 91 | Obp2b | odorant binding protein 2B | 1370632_at | 24.41 |
| 92 | EST | EST | 1381247_at | 24.40 |
| 93 | Ppp2r2b | protein phosphatase 2 (formerly 2A), regulatory subunit B (PR 52), beta isoform | 1387803_at | 24.02 |
| 94 | EST | EST | 1397393_at | 23.94 |
| 95 | Omd | osteomodulin | 1387197_at | 23.88 |
| 96 | LOC286989 | UDP-glucuronosyltransferase | 1370615_at | 23.86 |
| 97 | EST | EST | 1385780_at | 23.80 |
| 98 | EST | EST | 1395585_at | 23.70 |
| 99 | C4bpa | complement component 4 binding protein, alpha | 1369764_at | 23.59 |
| 100 | EST | EST | 1385065_at | 23.45 |
| 101 | Cd209g | CD209g molecule | 1384905_at | 23.42 |
| 102 | Igh-1a | immunoglobulin heavy chain 1a (serum IgG2a) | 1388272_at | 23.29 |
| 103 | EST | EST | 1394837_at | 23.22 |
| 104 | Cyp17a1 | cytochrome P450, family 17, subfamily a, polypeptide 1 | 1387123_at | 23.11 |
| 105 | Aqp4 | aquaporin 4 | 1368981_at | 23.05 |
| 106 | EST | EST | 1379786_at | 23.00 |
| 107 | EST | EST | 1397857_at | 22.83 |
| 108 | EST | EST | 1379517_at | 22.74 |
| 109 | Andpro | androgen regulated 20 kDa protein | 1388302_x_at | 22.71 |
| 110 | Lepr | leptin receptor | 1371223_a_at | 22.60 |
| 111 | EST | EST | 1388592_at | 22.53 |
| 112 | RGD1308215 | similar to hypothetical protein DKFZp434I2117 | 1390334_at | 22.43 |
| 113 | Aqp4 | aquaporin 4 | 1372190_at | 22.30 |
| 114 | Hist1h2aa | histone cluster 1, H2aa | 1369893_at | 22.00 |
| 115 | RGD1565709 | similar to ovostatin-2 | 1383956_at | 22.00 |
| 116 | Ndrg2 | N-myc downstream regulated gene 2 | 1398111_at | 21.88 |
| 117 | EST | EST | 1379776_at | 21.86 |
| 118 | EST | EST | 1381649_at | 21.75 |
| 119 | Tnmd | tenomodulin | 1368237_at | 21.59 |
| 120 | LOC679974 / LOC680282 / Tceal3 | similar to transcription elongation factor A (SII)-like 3 / hypothetical protein LOC680282 / transcription elongation factor A (SII)-like 3 | 1378855_a_at | 21.57 |
| 121 | Smoc2 | SPARC related modular calcium binding 2 | 1385242_at | 21.55 |
| 122 | Ibsp | integrin binding sialoprotein | 1368416_at | 21.53 |
| 123 | Entpd4 | ectonucleoside triphosphate diphosphohydrolase 4 | 1396615_at | 21.38 |
| 124 | Adora1 | adenosine A1 receptor | 1370584_a_at | 20.97 |
| 125 | EST | EST | 1395798_at | 20.88 |
| 126 | Atpif1 / LOC680367 / LOC680406 | ATPase inhibitory factor 1 / similar to Urinary protein 3 precursor (RUP-3) / similar to Urinary protein 2 precursor (RUP-2) | 1370350_x_at | 20.80 |
| 127 | Tmem28 | transmembrane protein 28 | 1383800_at | 20.71 |
| 128 | Cadps | Ca2+-dependent secretion activator | 1390754_at | 20.70 |
| 129 | EST | EST | 1385973_at | 20.67 |
| 130 | Itga11 | integrin, alpha 11 | 1394824_at | 20.45 |
| 131 | EST | EST | 1392343_at | 20.32 |
| 132 | EST | EST | 1392226_at | 20.00 |
| 133 | LOC679958 | similar to CG10806-PB, isoform B | 1385589_at | 20.00 |
| 134 | EST | EST | 1392110_at | 20.00 |
| 135 | EST | EST | 1395564_at | 19.80 |
| 136 | Ogn | osteoglycin | 1390450_a_at | 19.72 |
| 137 | Trem2 | triggering receptor expressed on myeloid cells 2 | 1392759_at | 19.53 |
| 138 | Ogn | osteoglycin | 1383263_at | 19.40 |
| 139 | Cmbl | carboxymethylenebutenolidase homolog (Pseudomonas) | 1396103_at | 19.25 |
| 140 | EST | EST | 1394858_at | 19.21 |
| 141 | EST | EST | 1394635_at | 19.19 |
| 142 | LOC681066 | similar to Ferritin heavy chain (Ferritin H subunit) (Proliferation-inducing gene 15 protein) | 1376671_at | 19.00 |
| 143 | LOC680282 | hypothetical protein LOC680282 | 1378857_at | 18.86 |
| 144 | EST | EST | 1375097_at | 18.86 |
| 145 | Slc15a2 | solute carrier family 15 (H+/peptide transporter), member 2 | 1398255_at | 18.84 |
| 146 | EST | EST | 1395539_at | 18.77 |
| 147 | Zfp90 | zinc finger protein 90 | 1398134_at | 18.67 |
| 148 | EST | EST | 1391047_at | 18.64 |
| 149 | EST | EST | 1384247_at | 18.63 |
| 150 | EST | EST | 1396934_at | 18.58 |
| 151 | EST | EST | 1396874_at | 18.56 |
| 152 | Spink3 | serine peptidase inhibitor, Kazal type 3 | 1387967_at | 18.50 |
| 153 | Cldn11 | claudin 11 | 1369609_at | 18.43 |
| 154 | Iyd | iodotyrosine deiodinase | 1384628_at | 18.30 |
| 155 | EST | EST | 1390713_at | 18.26 |
| 156 | EST | EST | 1396427_at | 18.17 |
| 157 | Abca8a | ATP-binding cassette, sub-family A (ABC1), member 8a | 1390783_at | 18.09 |
| 158 | LOC302999 | similar to tripartite motif protein 32 | 1374356_at | 18.07 |
| 159 | EST | EST | 1382845_at | 18.06 |
| 160 | Egfl6 | EGF-like-domain, multiple 6 | 1393335_at | 18.05 |
| 161 | EST | EST | 1393732_at | 18.05 |
| 162 | Ogn | osteoglycin | 1376749_at | 18.04 |
| 163 | Mamdc2 | MAM domain containing 2 | 1375983_at | 17.93 |
| 164 | Snap91 | synaptosomal-associated protein 91 | 1373865_at | 17.81 |
| 165 | Glra2 | glycine receptor, alpha 2 subunit | 1387696_a_at | 17.81 |
| 166 | Lrrc18 | leucine rich repeat containing 18 | 1393462_at | 17.77 |
| 167 | Sult1a1 | sulfotransferase family 1A, phenol-preferring, member 1 | 1370019_at | 17.64 |
| 168 | EST | EST | 1395290_at | 17.60 |
| 169 | rCG_34031 | similar to kynurenine formamidase | 1396301_x_at | 17.60 |
| 170 | Lemd2 | LEM domain containing 2 | 1376508_at | 17.57 |
| 171 | Bucs1 | Butyryl Coenzyme A synthetase 1 | 1384499_at | 17.42 |
| 172 | Lep | leptin | 1387748_at | 17.34 |
| 173 | Prl3c1 | Prolactin family 3, subfamily c, member 1 | 1368107_at | 17.33 |
| 174 | EST | EST | 1380005_at | 17.30 |
| 175 | EST | EST | 1382549_at | 17.26 |
| 176 | EST | EST | 1379496_at | 17.19 |
| 177 | Dmrt2 | doublesex and mab-3 related transcription factor 2 | 1384487_at | 17.18 |
| 178 | Itga7 | integrin alpha 7 | 1371214_at | 17.14 |
| 179 | Gal | galanin | 1387088_at | 17.12 |
| 180 | EST | EST | 1378148_at | 17.05 |
| 181 | Fmod | fibromodulin | 1367700_at | 17.02 |
| 182 | EST | EST | 1391225_at | 17.00 |
| 183 | RGD1561849 | similar to RIKEN cDNA 3110035E14 | 1390317_at | 17.00 |
| 184 | EST | EST | 1394417_at | 16.87 |
| 185 | EST | EST | 1380986_at | 16.78 |
| 186 | EST | EST | 1381333_at | 16.76 |
| 187 | EST | EST | 1383449_at | 16.75 |
| 188 | EST | EST | 1380884_at | 16.75 |
| 189 | Sv2b | synaptic vesicle glycoprotein 2b | 1369627_at | 16.74 |
| 190 | Gria2 | glutamate receptor, ionotropic, AMPA 2 | 1387171_at | 16.73 |
| 191 | Ankrd34b | ankyrin repeat domain 34B | 1393283_at | 16.73 |
| 192 | RGD1564722 | similar to C19orf36 protein | 1380573_a_at | 16.69 |
| 193 | EST | EST | 1379114_at | 16.64 |
| 194 | EST | EST | 1398229_at | 16.63 |
| 195 | EST | EST | 1395113_at | 16.59 |
| 196 | EST | EST | 1396966_at | 16.59 |
| 197 | EST | EST | 1380353_at | 16.57 |
| 198 | EST | EST | 1396374_at | 16.55 |
| 199 | Ogn | osteoglycin | 1385248_a_at | 16.54 |
| 200 | EST | EST | 1379105_at | 16.44 |

Table C. Genes up-regulated in the skin and subcutaneous tissue revealed by microarray analysis at 3 days after compression.

|  | Gene Symbol | Gene Name | Probe Set ID | Fold Change |
| --- | --- | --- | --- | --- |
|  |  |  |  | compression/sham |
| 1 | EST | EST | 1392809_at | 161.00 |
| 2 | EST | EST | 1379124_at | 109.00 |
| 3 | EST | EST | 1396931_at | 96.00 |
| 4 | LOC302022 | similar to nidogen 2 protein | 1381434_s_at | 65.90 |
| 5 | Sycp1 | synaptonemal complex protein 1 | 1387553_at | 59.50 |
| 6 | EST | EST | 1392395_at | 49.60 |
| 7 | EST | EST | 1382706_at | 47.67 |
| 8 | Hapln1 | hyaluronan and proteoglycan link protein 1 | 1384541_at | 47.50 |
| 9 | EST | EST | 1392255_at | 40.80 |
| 10 | EST | EST | 1383983_at | 40.00 |
| 11 | EST | EST | 1379837_at | 37.50 |
| 12 | RGD1307615 | similar to hypothetical protein FLJ13045 | 1380667_at | 37.09 |
| 13 | Eef1e1 / RGD1564159 | eukaryotic translation elongation factor 1 epsilon 1 / similar to eukaryotic translation elongation factor 1 epsilon 1 | 1395312_at | 35.00 |
| 14 | EST | EST | 1397451_at | 34.00 |
| 15 | EST | EST | 1378148_at | 33.50 |
| 16 | Mup5 | major urinary protein 5 | 1370778_at | 32.50 |
| 17 | EST | EST | 1380026_at | 32.00 |
| 18 | EST | EST | 1396366_at | 31.33 |
| 19 | EST | EST | 1372963_at | 30.33 |
| 20 | Gzmb | granzyme B | 1371126_x_at | 29.25 |
| 21 | EST | EST | 1384459_at | 28.65 |
| 22 | Nell1 | NEL-like 1 (chicken) | 1368120_at | 26.94 |
| 23 | Ero1lb | ERO1-like beta (S. cerevisiae) | 1377470_at | 26.78 |
| 24 | Lrrc19 | leucine rich repeat containing 19 | 1386671_at | 26.33 |
| 25 | EST | EST | 1395885_at | 26.00 |
| 26 | Usp49 | ubiquitin specific peptidase 49 | 1394150_at | 25.63 |
| 27 | Mrap | melanocortin 2 receptor accessory protein | 1379491_at | 25.15 |
| 28 | Ibsp | integrin binding sialoprotein | 1368416_at | 25.00 |
| 29 | Ifnk | interferon kappa | 1382073_at | 24.17 |
| 30 | EST | EST | 1394807_at | 23.07 |
| 31 | Apoa1 | apolipoprotein A-I | 1368335_at | 22.77 |
| 32 | EST | EST | 1390618_at | 22.58 |
| 33 | Kcna4 | potassium voltage-gated channel, shaker-related subfamily, member 4 | 1369043_at | 22.57 |
| 34 | EST | EST | 1386789_at | 22.29 |
| 35 | EST | EST | 1391631_at | 22.21 |
| 36 | Pyy | peptide YY (mapped) | 1393907_at | 21.86 |
| 37 | Cxcl3 | chemokine (C-X-C motif) ligand 3 | 1388033_at | 21.79 |
| 38 | EST | EST | 1377561_at | 21.75 |
| 39 | Chrm4 | cholinergic receptor, muscarinic 4 | 1388191_at | 21.64 |
| 40 | RGD1309095 | Similar to hypothetical protein BC015148 | 1395125_at | 21.40 |
| 41 | EST | EST | 1396454_at | 21.18 |
| 42 | EST | EST | 1396430_at | 20.80 |
| 43 | Umod | uromodulin | 1368333_at | 20.67 |
| 44 | Kcnc2 | potassium voltage gated channel, Shaw-related subfamily, member 2 | 1370558_a_at | 20.57 |
| 45 | EST | EST | 1375758_at | 20.44 |
| 46 | Birc7 | baculoviral IAP repeat-containing 7 (livin) | 1379215_at | 20.19 |
| 47 | Homer1 | homer homolog 1 (Drosophila) | 1370997_at | 20.00 |
| 48 | Cryga | crystallin, gamma A | 1374384_at | 20.00 |
| 49 | Nup62cl | nucleoporin 62 C-terminal like | 1398093_at | 19.67 |
| 50 | Grm1 | glutamate receptor, metabotropic 1 | 1371181_x_at | 19.61 |
| 51 | Lrrc23 | leucine rich repeat containing 23 | 1398465_at | 19.38 |
| 52 | EST | EST | 1395496_at | 19.15 |
| 53 | Spock3 | sparc/osteonectin, cwcv and kazal-like domains proteoglycan 3 | 1394252_at | 19.00 |
| 54 | Kcnh6 | potassium voltage-gated channel, subfamily H (eag-related), member 6 | 1369017_at | 18.94 |
| 55 | EST | EST | 1380905_at | 18.50 |
| 56 | Hsf2bp | heat shock transcription factor 2 binding protein | 1391722_at | 18.36 |
| 57 | Grk4 | G protein-coupled receptor kinase 4 | 1375594_at | 18.21 |
| 58 | EST | EST | 1396155_at | 18.17 |
| 59 | EST | EST | 1381647_at | 17.91 |
| 60 | Nrxn3 | neurexin 3 | 1381676_at | 17.82 |
| 61 | EST | EST | 1378852_at | 17.79 |
| 62 | RGD1306148 | Similar to KIAA0368 | 1378280_at | 17.70 |
| 63 | Art2b | ADP-ribosyltransferase 2b | 1382999_at | 17.43 |
| 64 | EST | EST | 1371751_at | 17.41 |
| 65 | Spinlw1 | serine protease inhibitor-like, with Kunitz and WAP domains 1 (eppin) | 1392781_at | 17.40 |
| 66 | EST | EST | 1397873_at | 17.33 |
| 67 | EST | EST | 1380204_at | 17.33 |
| 68 | Als2cr4 | amyotrophic lateral sclerosis 2 (juvenile) chromosome region, candidate 4 | 1381455_at | 17.12 |
| 69 | Cacnb4 | calcium channel, voltage-dependent, beta 4 subunit | 1371039_at | 17.09 |
| 70 | EST | EST | 1390337_at | 17.06 |
| 71 | EST | EST | 1397820_at | 16.54 |
| 72 | EST | EST | 1391816_at | 16.54 |
| 73 | EST | EST | 1396828_at | 16.50 |
| 74 | EST | EST | 1384666_at | 16.50 |
| 75 | EST | EST | 1392153_at | 16.13 |
| 76 | EST | EST | 1397720_at | 16.00 |
| 77 | EST | EST | 1397150_at | 15.89 |
| 78 | EST | EST | 1390069_at | 15.84 |
| 79 | Syt8 | synaptotagmin VIII | 1387842_at | 15.84 |
| 80 | EST | EST | 1398461_at | 15.68 |
| 81 | EST | EST | 1391735_at | 15.62 |
| 82 | EST | EST | 1397865_at | 15.57 |
| 83 | EST | EST | 1391090_at | 15.55 |
| 84 | RY2G5 | potential ligand-binding protein | 1384547_at | 15.43 |
| 85 | EST | EST | 1385782_at | 15.05 |
| 86 | EST | EST | 1373720_at | 15.00 |
| 87 | EST | EST | 1393789_at | 14.92 |
| 88 | Casc3 | cancer susceptibility candidate 3 | 1394704_at | 14.91 |
| 89 | EST | EST | 1384061_at | 14.82 |
| 90 | Kcnh8 | potassium voltage-gated channel, subfamily H (eag-related), member 8 | 1388042_at | 14.74 |
| 91 | Gnaz | guanine nucleotide binding protein, alpha z subunit | 1368185_at | 14.67 |
| 92 | EST | EST | 1375837_at | 14.64 |
| 93 | Bhmt2 | betaine-homocysteine methyltransferase 2 | 1391417_at | 14.64 |
| 94 | Cpeb3 | Cytoplasmic polyadenylation element binding protein 3 | 1381851_at | 14.62 |
| 95 | EST | EST | 1394867_at | 14.43 |
| 96 | EST | EST | 1394615_at | 14.42 |
| 97 | EST | EST | 1380413_at | 14.19 |
| 98 | EST | EST | 1375103_at | 14.15 |
| 99 | Sult1c2 | sulfotransferase family, cytosolic, 1C, member 2 | 1377672_at | 14.00 |
| 100 | EST | EST | 1377220_at | 13.88 |
| 101 | EST | EST | 1377529_at | 13.67 |
| 102 | EST | EST | 1375195_at | 13.60 |
| 103 | RGD1306534 | Similar to P-Rex1 | 1392436_at | 13.43 |
| 104 | EST | EST | 1373714_at | 13.43 |
| 105 | Lhfpl3 | lipoma HMGIC fusion partner-like 3 | 1383419_at | 13.41 |
| 106 | Exosc6 | Exosome component 6 | 1386623_at | 13.36 |
| 107 | EST | EST | 1394835_at | 13.20 |
| 108 | Lrrc56 | leucine rich repeat containing 56 | 1391087_at | 13.16 |
| 109 | EST | EST | 1377976_at | 13.16 |
| 110 | Tnr | tenascin R | 1369692_at | 13.00 |
| 111 | EST | EST | 1385371_at | 13.00 |
| 112 | Cyp2c6 / LOC293989 | cytochrome P450, subfamily IIC6 / cytochrome P450-like | 1370580_a_at | 13.00 |
| 113 | LOC501046 | similar to Phakinin (Beaded filament structural protein 2) (Lens fiber cell beaded filament protein CP 49) (CP49) (49 kDa cytoskeletal protein) | 1383812_at | 12.94 |
| 114 | Lgi1 | leucine-rich repeat LGI family, member 1 | 1386023_at | 12.94 |
| 115 | EST | EST | 1397595_at | 12.93 |
| 116 | Slco1a6 | solute carrier organic anion transporter family, member 1a6 | 1369401_at | 12.89 |
| 117 | EST | EST | 1381370_at | 12.87 |
| 118 | EST | EST | 1392783_at | 12.85 |
| 119 | EST | EST | 1398707_at | 12.73 |
| 120 | Vegp1 | von Ebners gland protein 1 | 1387246_at | 12.73 |
| 121 | Rab17 | RAB17, member RAS oncogene family | 1393319_a_at | 12.71 |
| 122 | Mcpt8 | mast cell protease 8 | 1369586_at | 12.71 |
| 123 | EST | EST | 1395179_at | 12.67 |
| 124 | EST | EST | 1380398_at | 12.54 |
| 125 | Tshz1 | Similar to teashirt family zinc finger 1 | 1392611_at | 12.45 |
| 126 | EST | EST | 1377942_at | 12.42 |
| 127 | Srcrb4d | scavenger receptor cysteine rich domain containing, group B (4 domains) | 1398141_at | 12.39 |
| 128 | Gucy1a2 | guanylate cyclase 1, soluble, alpha 2 | 1368974_at | 12.24 |
| 129 | EST | EST | 1385312_at | 12.03 |
| 130 | EST | EST | 1386433_at | 12.00 |
| 131 | EST | EST | 1397260_at | 12.00 |
| 132 | Rasgef1c | RasGEF domain family, member 1C | 1389811_at | 11.84 |
| 133 | Fgfr2 | fibroblast growth factor receptor 2 | 1375129_at | 11.67 |
| 134 | EST | EST | 1395942_at | 11.67 |
| 135 | Rcvrn | recoverin | 1369140_at | 11.67 |
| 136 | EST | EST | 1371275_at | 11.65 |
| 137 | Grid1 | glutamate receptor, ionotropic, delta 1 | 1369945_at | 11.63 |
| 138 | EST | EST | 1396983_at | 11.60 |
| 139 | EST | EST | 1384419_at | 11.59 |
| 140 | EST | EST | 1397640_at | 11.59 |
| 141 | Btg4 | B-cell translocation gene 4 | 1385736_at | 11.55 |
| 142 | EST | EST | 1392366_at | 11.55 |
| 143 | EST | EST | 1378496_at | 11.50 |
| 144 | Gad1 | glutamic acid decarboxylase 1 | 1368344_at | 11.50 |
| 145 | RGD1311874 | hypothetical LOC300751 | 1372387_at | 11.49 |
| 146 | C5 | complement component 5 | 1383425_at | 11.42 |
| 147 | Trfr2 | transferrin receptor 2 | 1380066_at | 11.38 |
| 148 | EST | EST | 1385703_at | 11.30 |
| 149 | EST | EST | 1382876_at | 11.27 |
| 150 | EST | EST | 1381330_at | 11.25 |
| 151 | Keg1 | kidney expressed gene 1 | 1370592_at | 11.22 |
| 152 | Ighe | immunoglobulin heavy chain (epsilon polypeptide) | 1371118_a_at | 11.18 |
| 153 | EST | EST | 1380840_at | 11.13 |
| 154 | EST | EST | 1382712_at | 11.07 |
| 155 | EST | EST | 1385997_at | 11.07 |
| 156 | EST | EST | 1383039_at | 11.04 |
| 157 | Znf512b | Zinc finger protein 512B | 1379852_at | 11.00 |
| 158 | EST | EST | 1386109_at | 10.93 |
| 159 | EST | EST | 1397116_at | 10.86 |
| 160 | Cfh / Cfhl1 | complement component factor H / complement component factor h-like 1 | 1382678_at | 10.86 |
| 161 | EST | EST | 1396269_at | 10.85 |
| 162 | Kcnip1 | Kv channel-interacting protein 1 | 1370781_a_at | 10.84 |
| 163 | EST | EST | 1384646_at | 10.83 |
| 164 | EST | EST | 1396868_at | 10.81 |
| 165 | EST | EST | 1396110_at | 10.80 |
| 166 | Fgd6 | FYVE, RhoGEF and PH domain containing 6 | 1375588_at | 10.80 |
| 167 | Thra | thyroid hormone receptor alpha | 1380518_s_at | 10.71 |
| 168 | LOC683844 / LOC689589 / Rnf11l | similar to RING finger protein 11 (NEDD4 WW domain-binding protein 2) (Sid 1669) / hypothetical protein LOC689589 / ring finger protein 11-like | 1398650_at | 10.69 |
| 169 | EST | EST | 1376960_at | 10.63 |
| 170 | Camk2n1 | calcium/calmodulin-dependent protein kinase II inhibitor 1 | 1370853_at | 10.62 |
| 171 | EST | EST | 1396607_at | 10.61 |
| 172 | EST | EST | 1373762_a_at | 10.50 |
| 173 | Fgb | fibrinogen, B beta polypeptide | 1388266_at | 10.50 |
| 174 | Gpr50 | G protein-coupled receptor 50 | 1384979_at | 10.49 |
| 175 | EST | EST | 1391605_at | 10.47 |
| 176 | EST | EST | 1385278_at | 10.47 |
| 177 | EST | EST | 1396400_at | 10.45 |
| 178 | EST | EST | 1390971_at | 10.41 |
| 179 | Rgs17 | regulator of G-protein signaling 17 | 1394280_at | 10.38 |
| 180 | EST | EST | 1394581_at | 10.38 |
| 181 | EST | EST | 1379114_at | 10.37 |
| 182 | Il27ra | interleukin 27 receptor, alpha | 1382652_at | 10.35 |
| 183 | Klf12 | Kruppel-like factor 12 | 1385545_at | 10.34 |
| 184 | Rnf207 | ring finger protein 207 | 1375931_at | 10.33 |
| 185 | EST | EST | 1381721_at | 10.30 |
| 186 | EST | EST | 1385664_at | 10.30 |
| 187 | EST | EST | 1376618_at | 10.27 |
| 188 | Kcnj10 | potassium inwardly-rectifying channel, subfamily J, member 10 | 1387467_at | 10.27 |
| 189 | EST | EST | 1394582_at | 10.26 |
| 190 | EST | EST | 1391799_at | 10.25 |
| 191 | EST | EST | 1396777_at | 10.21 |
| 192 | Pou3f4 | POU domain, class 3, transcription factor 4 | 1387677_at | 10.20 |
| 193 | EST | EST | 1378704_at | 10.19 |
| 194 | EST | EST | 1397968_at | 10.14 |
| 195 | EST | EST | 1378554_at | 10.13 |
| 196 | EST | EST | 1384694_at | 10.11 |
| 197 | Folh1 | folate hydrolase | 1387364_at | 10.07 |
| 198 | EST | EST | 1395138_at | 10.05 |
| 199 | LOC685001 | Similar to MIR-interacting saposin-like protein precursor (Transmembrane protein 4) (Putative secreted protein ZSIG9) | 1382918_at | 10.03 |
| 200 | EST | EST | 1393509_at | 9.96 |
