# Supplementary material for: Analysis of Gene Expression in Experimental Pressure Ulcers in the Rat with Special Reference to Inflammatory Cytokines
Source: PLoS One. 2015 Jul 15;10(7):e0132622. doi: 10.1371/journal.pone.0132622 (PMC4503587; doi:10.1371/journal.pone.0132622)
Supplement: S2 File — The values are at 12 hours (Table A), at 1 day (Table B), and 3 days (Table C) after compression. Biological process classified by gene ontology (obtained from NetAffx Analysis Center) are represented at left column, and the number of genes which increased over twice. (DOCX) [file pone.0132622.s002.docx]

Table A. Classification of up-regulated genes at 12 hours after compression.

| Category | Numbers of  up-regulated genes |
| --- | --- |
| transport | 188 |
| regulation of transcription, DNA-dependent | 171 |
| signal transduction | 164 |
| response to drug | 85 |
| metabolic process | 84 |
| positive regulation of transcription from RNA polymerase II promoter | 74 |
| immune response | 72 |
| apoptosis | 63 |
| proteolysis | 58 |
| inflammatory response | 54 |
| positive regulation of apoptosis | 53 |
| negative regulation of apoptosis | 50 |
| response to hypoxia | 49 |
| cell proliferation | 48 |
| response to lipopolysaccharide | 40 |
| response to organic cyclic substance | 40 |
| anti-apoptosis | 38 |
| aging | 35 |
| negative regulation of cell proliferation | 34 |
| response to organic substance | 33 |
| spermatogenesis | 32 |
| regulation of cell proliferation | 31 |
| angiogenesis | 29 |
| response to oxidative stress | 29 |
| response to glucocorticoid stimulus | 27 |
| chemotaxis | 26 |
| defense response to bacterium | 22 |
| ossification | 21 |
| response to cytokine stimulus | 21 |
| female pregnancy | 20 |
| positive regulation of transcription, DNA-dependent | 20 |
| cellular calcium ion homeostasis | 19 |
| neutrophil chemotaxis | 19 |
| wound healing | 19 |
| cytokine-mediated signaling pathway | 18 |
| positive regulation of smooth muscle cell proliferation | 18 |
| extracellular matrix organization | 17 |
| response to hydrogen peroxide | 17 |
| response to ethanol | 16 |
| skeletal system development | 16 |
| neuropeptide signaling pathway | 15 |
| response to cAMP | 15 |
| response to heat | 15 |
| response to mechanical stimulus | 14 |
| positive regulation of anti-apoptosis | 13 |
| response to nutrient | 13 |
| cell growth | 12 |
| cellular response to extracellular stimulus | 12 |
| neuron projection development | 12 |
| positive regulation of MAPKKK cascade | 12 |
| acute-phase response | 11 |
| glucose homeostasis | 11 |
| positive regulation of peptidyl-tyrosine phosphorylation | 10 |
| positive regulation of cell cycle | 9 |
| positive regulation of NF-kappaB transcription factor activity | 9 |
| response to corticosterone stimulus | 9 |
| tissue regeneration | 9 |
| circadian rhythm | 8 |
| leukocyte chemotaxis | 8 |
| neutrophil apoptosis | 8 |
| response to radiation | 8 |
| collagen catabolic process | 7 |
| electron transport chain | 7 |
| positive regulation of epithelial cell proliferation | 7 |
| positive regulation of interleukin-6 production | 7 |
| positive regulation of T cell proliferation | 7 |
| response to progesterone stimulus | 7 |
| transforming growth factor beta receptor signaling pathway | 7 |
| learning | 6 |
| positive regulation of chemokine production | 6 |
| positive regulation of endothelial cell proliferation | 6 |
| positive regulation of synaptic transmission | 6 |
| cytokine production | 5 |
| defense response to Gram-positive bacterium | 5 |
| endocrine pancreas development | 5 |
| positive regulation of smooth muscle contraction | 5 |
| positive regulation of translation | 5 |
| response to bacterium | 5 |
| response to reactive oxygen species | 5 |
| response to vitamin A | 5 |
| rhythmic process | 5 |
| vascular endothelial growth factor receptor signaling pathway | 5 |
| defense response to Gram-negative bacterium | 4 |
| defense response to protozoan | 4 |
| defense response to virus | 4 |
| interleukin-6-mediated signaling pathway | 4 |
| lymphocyte chemotaxis | 4 |
| macrophage chemotaxis | 4 |
| positive regulation of tyrosine phosphorylation of Stat3 protein | 4 |
| muscle maintenance | 3 |
| negative regulation of proteolysis | 3 |
| positive regulation of keratinocyte migration | 3 |
| positive regulation of synaptic plasticity | 3 |
| positive regulation of T-helper 2 cell differentiation | 3 |
| regulation of angiogenesis | 3 |
| response to gravity | 3 |
| salivary gland morphogenesis | 3 |
| tissue remodeling | 3 |
| chemokine-mediated signaling pathway | 2 |
| chemokinesis | 2 |
| chronological cell aging | 2 |
| negative regulation of hormone secretion | 2 |
| positive regulation of leukocyte migration | 2 |
| positive regulation of peptidyl-serine phosphorylation | 2 |
| positive regulation of transcription from RNA polymerase II promoter, mitotic | 2 |
| regulation of circadian sleep/wake cycle, sleep | 2 |
| response to peptidoglycan | 2 |
| detection of bacterium | 1 |
| hepatic immune response | 1 |
| monocyte chemotaxis | 1 |
| negative regulation of chemokine biosynthetic process | 1 |
| negative regulation of collagen biosynthetic process | 1 |
| peptide hormone secretion | 1 |
| peptidoglycan catabolic process | 1 |
| positive regulation of acute inflammatory response | 1 |
| positive regulation of B cell activation | 1 |
| positive regulation of immunoglobulin secretion | 1 |
| positive regulation of JAK-STAT cascade | 1 |
| regulation of circadian sleep/wake cycle, non-REM sleep | 1 |
| regulation of vascular endothelial growth factor production | 1 |

Table B. Classification of up-regulated genes at 1 day after compression.

| Category | Numbers of  up-regulated genes |
| --- | --- |
| transport | 237 |
| metabolic process | 126 |
| proteolysis | 103 |
| cell adhesion | 102 |
| phosphate transport | 39 |
| carbohydrate metabolic process | 37 |
| ossification | 32 |
| visual perception | 27 |
| cell-cell adhesion | 17 |
| digestion | 15 |
| extracellular matrix organization and biogenesis | 14 |
| collagen fibril organization | 7 |
| cartilage condensation | 6 |
| protein-chromophore linkage | 5 |
| proteoglycan metabolic process | 4 |
| bioluminescence | 2 |

Table C. Classification of up-regulated genes at 3 days after compression.

| Category | Numbers of  up-regulated genes |
| --- | --- |
| transport | 155 |
| proteolysis | 48 |
| cell adhesion | 29 |
| small GTPase mediated signal transduction | 13 |
| spermatogenesis | 9 |
| translation | 8 |
| cell cycle | 7 |
| cytolysis | 5 |
| cell division | 3 |
| cell-matrix adhesion | 3 |
| induction of apoptosis by granzyme | 3 |
| meiosis | 3 |
| synaptonemal complex assembly | 2 |
| meiotic recombination | 1 |
