# Supplementary material for: Analysis of Gene Expression in Experimental Pressure Ulcers in the Rat with Special Reference to Inflammatory Cytokines
Source: PLoS One. 2015 Jul 15;10(7):e0132622. doi: 10.1371/journal.pone.0132622 (PMC4503587; doi:10.1371/journal.pone.0132622)
Supplement: S3 File — RNAs from the skin and the subcutaneous tissue analyzed. Up-pointing arrows: up-regulated over twice, down-pointing arrows: down-regulated less than half. (DOCX) [file pone.0132622.s003.docx]

Table. Results of Microarray analysis with receptors to inflammatory cytokine.

|  | Abbreviation | Gene Name | Probe ID | 12h | |  | 1d | |  | 3d | |
| --- | --- | --- | --- | --- | --- | --- | --- | --- | --- | --- | --- |
|  |  |  |  | n=1 | |  | n=1 | |  | n=1 | |
| GM-CSF receptor | Csf2ra | Granulocyte-macrophage colony stimulating receptor alpha | 1392528_at | 2.00 | ↑ |  | 0.66 |  |  | 0.50 | ↓ |
|  | Csf2rb | colony stimulating factor 2 receptor, beta, low-affinity (granulocyte-macrophage) | 1369828_at | 4.39 | ↑ |  | 0.51 |  |  | 0.94 |  |
| IFN-γ receptor | Ifngr1 | interferon gamma receptor 1 | 1369956_at | 1.59 |  |  | 1.00 |  |  | 0.95 |  |
|  | Ifngr2 | interferon gamma receptor 2 | 1374601_at | 2.21 | ↑ |  | 0.64 |  |  | 1.02 |  |
| IL-1 receptor (IL1Ra receptor) | Il1r1 | interleukin 1 receptor, type I | 1369255_at | 2.65 | ↑ |  | 1.46 |  |  | 0.40 | ↓ |
|  | Il1r1 | interleukin 1 receptor, type I | 1370750_a_at | 5.01 | ↑ |  | 0.50 |  |  | 1.08 |  |
|  | Il1r2 | interleukin 1 receptor, type II | 1387180_at | 2.10 | ↑ |  | 3.80 | ↑ |  | 1.71 |  |
| IL-2 receptor | Il2ra | interleukin 2 receptor, alpha | 1387591_at | 0.77 |  |  | 0.86 |  |  | 1.15 |  |
|  | Il2rb | interleukin 2 receptor, beta | 1387394_at | 1.03 |  |  | 0.95 |  |  | 1.11 |  |
| IL-6 receptor | Il6ra | interleukin 6 receptor, alpha | 1386987_at | 2.17 | ↑ |  | 1.17 |  |  | 0.83 |  |
| IL-10 receptor | Il10ra | interleukin 10 receptor, alpha | 1387728_at | 1.30 |  |  | 1.17 |  |  | 1.46 |  |
|  | Il10rb | interleukin 10 receptor, beta | 1383616_at | 1.33 |  |  | 0.85 |  |  | 0.71 |  |
| IL-11 receptor | Il11ra1 | interleukin 11 receptor, alpha chain 1 | 1370331_at | 0.31 | ↓ |  | 3.21 | ↑ |  | 1.53 |  |
| LT-β receptor | Ltbr | lymphotoxin beta receptor (TNFR superfamily, member 3) | 1372914_at | 1.51 |  |  | 0.93 |  |  | 0.76 |  |
| TGF-β receptor | Tgfbr1 | transforming growth factor, beta receptor 1 | 1369504_at | 1.48 |  |  | 0.92 |  |  | 1.40 |  |
|  | Tgfbr1 | transforming growth factor, beta receptor 1 | 1376636_at | 0.95 |  |  | 0.88 |  |  | 0.85 |  |
|  | Tgfbr2 | transforming growth factor, beta receptor II | 1369653_at | 1.59 |  |  | 1.56 |  |  | 0.83 |  |
|  | Tgfbr3 | transforming growth factor, beta receptor III | 1369219_at | 0.75 |  |  | 1.67 |  |  | 1.03 |  |
|  | Tgfbr3 | transforming growth factor, beta receptor III | 1387484_at | 0.52 |  |  | 2.24 | ↑ |  | 1.02 |  |
| TNF receptor | Tnfrsf1a | tumor necrosis factor receptor superfamily, member 1a | 1367715_at | 2.16 | ↑ |  | 0.78 |  |  | 0.95 |  |
|  | Tnfrsf1b | tumor necrosis factor receptor superfamily, member 1b | 1392731_at | 48.42 | ↑ |  | 0.55 |  |  | 0.76 |  |
|  | Tnfrsf4 | tumor necrosis factor receptor superfamily, member 4 | 1387621_at | 0.16 | ↓ |  | 1.81 |  |  | 0.36 | ↓ |
|  | Tnfrsf8 | tumor necrosis factor receptor superfamily, member 8 | 1368635_at | 0.43 | ↓ |  | 2.39 | ↑ |  | 1.89 |  |
|  | Tnfrsf9 | tumor necrosis factor receptor superfamily, member 9 | 1375900_at | 2.16 | ↑ |  | 0.58 |  |  | 0.63 | ↓ |
|  | Tnfrsf11a | tumor necrosis factor receptor superfamily, member 11a | 1394313_at | 1.61 |  |  | 0.27 | ↓ |  | 0.72 |  |
|  | Tnfrsf12a | tumor necrosis factor receptor superfamily, member 12a | 1371785_at | 8.31 | ↑ |  | 0.23 | ↓ |  | 0.39 |  |
|  | Tnfrsf14 | tumor necrosis factor receptor superfamily, member 14 (herpesvirus entry mediator) | 1376327_at | 3.95 | ↑ |  | 0.53 |  |  | 0.08 | ↓ |
|  | Tnfrsf17 | tumor necrosis factor receptor superfamily, member 17 | 1384441_at | 4.55 | ↑ |  | 1.32 |  |  | 3.97 | ↑ |
|  | Tnfrsf17 | tumor necrosis factor receptor superfamily, member 17 | 1385710_at | 1.56 |  |  | 0.69 |  |  | 1.25 |  |
|  | Tnfrsf21 | tumor necrosis factor receptor superfamily, member 21 | 1391573_at | 1.42 |  |  | 0.51 |  |  | 0.99 |  |
|  | Tnfrsf21 | tumor necrosis factor receptor superfamily, member 21 | 1393584_at | 1.75 |  |  | 0.47 | ↓ |  | 0.94 |  |
|  | Tnfrsf25 | tumor necrosis factor receptor superfamily, member 25 | 1393866_at | 0.79 |  |  | 0.94 |  |  | 1.16 |  |
|  | Tnfrsf26 | tumor necrosis factor receptor superfamily, member 26 | 1395635_at | 3.60 | ↑ |  | 0.39 | ↓ |  | 1.17 |  |
